# Neural network prediction model based on Levy flight and natural biomimetic technology for its application in cancer prediction

**DOI:** 10.1371/journal.pone.0326874

**Published:** 2025-06-25

**Authors:** Ruiyu Zhan

**Affiliations:** Department of Oncology, Zigong Fourth People’s Hospital, Zigong, China; University 20 Aout 1955 skikda, Algeria, ALGERIA

## Abstract

Precise forecasting of cancer outcomes is essential for medical professionals to assess the well-being of patients and develop customized therapeutic plans. Despite its importance, achieving precise forecasts remains a formidable challenge. To tackle this issue, we present an innovative method that harmonizes the Grey Wolf Optimizer (GWO) with Levy flight to optimize the weights and biases of a Backpropagation (BP) neural network—a prominent machine learning model extensively employed in classification tasks. Our novel approach, LGWO-BP, is tailored to augment the precision of cancer prognosis predictions. We performed comparative analyses against other methodologies across various functions and public datasets to assess their effectiveness. The experimental results show the exceptional strengths of the proposed LGWO-BP method, particularly its accuracy and reliability compared to GWO-BP, and show that it achieves comparative results against state-of-the-art (SOTA) methods. Our assessment of the LGWO-BP technique’s efficacy involved undertaking empirical tests across half a dozen openly accessible datasets. For the early-stage diabetes dataset, LGWO-BP achieved an accuracy of 0.92, a recall of 0.93, a precision of 0.88, an F1-score of 0.91, and an AUC of 0.95. Utilizing the diabetes dataset from 130 U.S. hospitals, the LGWO-BP algorithm achieved a precision rate of 0.97, a sensitivity of 1.00, a correct classification rate of 0.99, a harmonic mean of precision and recall (F1-score) of 0.98, and an area under the ROC curve (AUC) of 1.00. For the diabetes health indicators dataset, LGWO-BP achieved an accuracy of 0.9 and an AUC of 1. Leveraging data from The Cancer Genome Atlas (TCGA) — a U.S.-led initiative conducting in-depth molecular research to elucidate the causative mechanisms of cancer — this study focuses on three specific cancer types within the dataset: lung, breast, and esophageal cancers. TCGA provides a rich repository of genomic, transcriptomic, epigenomic, and patient-specific clinical data across 33 cancer types. In evaluating the prognostic performance of the LGWO-BP (Lévy flight-enhanced Grey Wolf Optimizer integrated with Back Propagation) model, we observed AUC (Area Under the Curve) scores of 0.70 for miRNA expression, 0.72 for gene expression, and 0.72 for DNA methylation. Regarding precision, the model achieved accuracies of 0.67, 0.69, and 0.66 for miRNA expression, gene expression, and DNA methylation, respectively. For recall, the corresponding values were 0.71, 0.61, and 0.62. Notably, the F1-scores, which balance precision and recall, were 0.69 for miRNA expression, 0.65 for gene expression, and 0.62 for DNA methylation. This research not only advances the application of machine learning in medical prognosis but also offers crucial guidance for clinicians in developing more precise and reliable prognostic tools for cancer patients. By enhancing the efficacy of machine learning-driven cancer prognosis, our proposed LGWO-BP approach has the potential to improve patient care and treatment outcomes significantly.

## 1. Introduction

Cancer is a threat that surrounds humans every moment, and research on cancer has always been of great interest [1]. It is vital to precisely distinguish between aggressive and chronic forms of cancer to forecast patient prognoses and guide pivotal therapeutic decisions. Risk prediction predominantly relies on the appraisal of the disease condition via histopathological and radiological assessments [2]. Attributes such as the progression of cancer into lymphatic nodes and the poor differentiation of cells are considered key predictors of patient outcomes and are utilized in determining the cancer’s stage and grade. Nevertheless, these pathological markers are subjective and exhibit low observer consistency. Despite precise classification of the tumor stages, accurate foresight into the patient’s clinical course is unattainable [3]. Current prognostic methods, despite their advancements, still face challenges in achieving high accuracy and consistency due to the subjective nature of pathological markers and the complexity of cancer progression. This underscores the need for more sophisticated and robust prognostic tools that can overcome these limitations.

In addition to traditional methods based on tumor staging and pathological type judgment to predict prognosis, methods based on copy number determination, DNA sequencing, gene expression analysis, and other genomic technologies have emerged for prognosis analysis. By using these innovative methods, disease prognosis can be predicted and specific treatment plans can be guided. The initiative dubbed The Cancer Genome Atlas(TCGA) has amassed genetic and clinical data on thirty-three distinct cancer types, offering a considerable repository for the identification of biomarkers [4]. At present, the primary focus of endeavors aimed at identifying predictive biomarkers lies in detecting alterations in gene expression that correlate with clinical outcomes [5]. In the field of machine learning-based precise cancer prognosis, various methods such as random forest, multi-layer perceptron, and SVM, have been employed for predicting cancer outcomes [6]. For instance, Suleyman et al. Utilizing a quintet of methods, [7] forecasted the prognoses for breast cancer in a cohort of 358 individuals from the TCGA database, based on their somatic mutation profiles. Random forest achieved the highest accuracy 0.70, but overall results were unsatisfactory. Despite their resilience when handling data with many dimensions, random forests are not without shortcomings; they are prone to overfitting on the training set [8]. In another study [9], Yuan et al. classified lung cancer subtypes using random forest and SVM, alongside feature selection techniques. Higher accuracy was observed with more features, achieving 0.96 with SVM and 0.93 with RF, dropping when fewer features were used. SVM excels in high-dimensional spaces. Nevertheless, the performance of Support Vector Machines can be considerably impacted by the presence of incomplete data, resulting in potentially imprecise forecasts unless managed correctly [8]. Multi-layer perceptron networks capture complex patterns but require careful tuning and may get stuck in local optima [9]. A study on lung adenocarcinoma gene expression data identified potential biomarkers. Classification using these biomarkers yielded accuracies of 0.87 for MLP [10]. However, more scrutiny is needed to improve clarity and precision, as current models often overlook the interplay between genetic markers and clinical outcomes, hindering optimal cancer prediction

Contemporary health studies have sparked an increased fascination with refinement techniques for choosing attributes, particularly focused on improving algorithms for machine learning. These techniques refine data features to improve classifier accuracy and overall model performance [11,12]. Researchers such as El-Hassani et al. and Maja Guberina et al.Utilized algorithmic models rooted in machine learning for predictive analysis of both hypothyroidism and cancerous lymph node involvement in the context of non-small cell lung cancer [13,14]. Sam Khozama introduced a novel breast cancer prediction model grounded in Bayes’ Theorem and ensemble learning, achieving high accuracy [15].

Ultimately, the progression of medical studies has employed the use of optimization algorithms, as well as machine and deep learning methods, to develop extremely accurate predictive models for numerous illnesses such as breast cancer, heart disease, and chronic kidney disease, with certain models reaching precision levels of over 95%. The emergence of innovative metaheuristic approaches like RBMO underscores the potential of optimization algorithms in resolving intricate medical and engineering challenges.

Additionally, the optimization algorithm known as Grey Wolf Optimizer (GWO), which draws its inspiration from the hunting patterns of grey wolves, has developed as a method grounded in the principles of swarm intelligence [16]. The integration of GWO with Levy flight capitalizes on their complementary strengths. While GWO effectively navigates the search space for global optima, Levy flight’s long jumps facilitate escaping local optima and exploring overlooked regions, fostering a more exhaustive and efficient search process. By incorporating machine learning, computational algorithms are honed through dataset training, enabling them to forecast or categorize unseen data [17].

Integrating Grey Wolf Optimization (GWO) with these machine learning techniques assists in disease forecasting by serving two key functions: Firstly, feature selection is enhanced by emulating grey wolf dynamics, leading to a more efficient and higher-quality set of features; Secondly, Fine-tuning Parameters: The process of tweaking parameter settings serves to refine and enhance the efficiency and precision of machine learning algorithms;3. Sample Selection: Modifying how samples are chosen in order to obtain the most indicative examples boosts the model’s ability to forecast accurately;4. Model Integration: Perfecting the combination and balance of multiple models to elevate the accuracy of predictions [18]. The integration of Levy flight into GWO specifically addresses the need for a more dynamic exploration strategy in the optimization process, which is crucial for complex and nonlinear problems like cancer prognosis prediction.

Integrating the Grey Wolf Optimizer with machine learning techniques enhances the efficiency and precision of forecasting frameworks, which proves advantageous in the anticipation and identification of medical conditions [19]. Despite these advances, the field remains rapidly evolving, with recent research exploring hybrid optimization strategies that combine the strengths of different algorithms to achieve even better performance. Incorporating Levy flight within the framework of the GWO algorithm, as this research suggests, represents an instance dedicated to surmounting the inherent constraints of conventional optimization methods.

In neural network training, the GWO algorithm may have higher performance than other metaheuristic algorithms and has already been applied in training BP models [20]. Nonetheless, the utilization of Grey Wolf Optimization for the purpose of neural network training, especially when it pertains to forecasting outcomes in cancer prognosis, is still to a great extent an untouched area of research. Moreover, the potential of combining GWO with other strategies, such as Levy flight, to further enhance prediction accuracy has not been fully investigated.

Recently, hybrid models that merge the benefits of gradient-based algorithms (simplicity, speed, and local optimal solutions) with metaheuristic algorithms (prevention of local optimal solutions) have gained popularity in neural network training [21]. Illustratively, certain research integrates the SHO and BP algorithm [22], while different studies merge the BP algorithm with the PO algorithm [23], and additional research pairs the BP algorithm with the PSO algorithm [24]. Additionally, the concept of Levy flight denotes a stochastic mechanism based on the principles of the Levy distribution [25]. By incorporating Levy flight into the GWO-BP framework, we aim to achieve a balance between the thoroughness of GWO’s global search and the efficiency of BP’s local optimization, thereby enhancing the overall performance for cancer prognosis prediction.

In this research, a novel GWO algorithm leveraging Levy flight is introduced. This approach incorporates Levy flight when the performance of the GWO algorithm plateaus after a specific number of iterations, enhancing its capabilities. The GWO algorithm excels in seeking global optimal solutions, while the BP algorithm is adept at locating local optimal results. The primary objective of this study is to integrate the LGWO with the BP neural network and maximize the benefits of both local and global search strategies.

Key outcomes of this research include:1) A principal advancement provided by this research is the enhancement of the GWO through the integration of Levy flight, which in turn bolsters the efficacy of the GWO algorithm.2) Utilizing genomic data across 33 types of cancer sourced from TCGA, coupled with corresponding clinical details, this investigation has pinpointed potential prognostic factors.The research validates the effectiveness and accuracy of the optimization method through the incorporation of the improved LGWO into the BP neural network and evaluates its ability to predict the results of cancer prognoses.

Importance: 1)The research incorporates Levy flight to enhance the GWO algorithm’s approach, effectively introducing the LGWO optimization algorithm. 2) Compared with the unimproved GWO algorithm, the LGWO algorithm demonstrates better performance during the optimization process, providing superior results for BP neural network classification tasks. 3) By using optimization algorithms for prediction, more accurate prognostic information can be provided to clinical doctors, helping to develop precise treatment plans, improve patient treatment outcomes, and positively impact cancer treatment quality.

This document is structured in the following manner: Section 2 examines pertinent scholarly works. Section 3 provides a detailed explanation of the LGWO algorithm and its procedural sequence. Section 4 details the results of simulated examinations along with an analysis of standard testing functions. Section 5 demonstrates the application of LGWO in various fundamental engineering tasks, such as cancer prognosis prediction. Lastly, Section 6 offers conclusions.

## 2. Related work

### 2.1. Grey wolf optimizer overview

**Fig 1:**
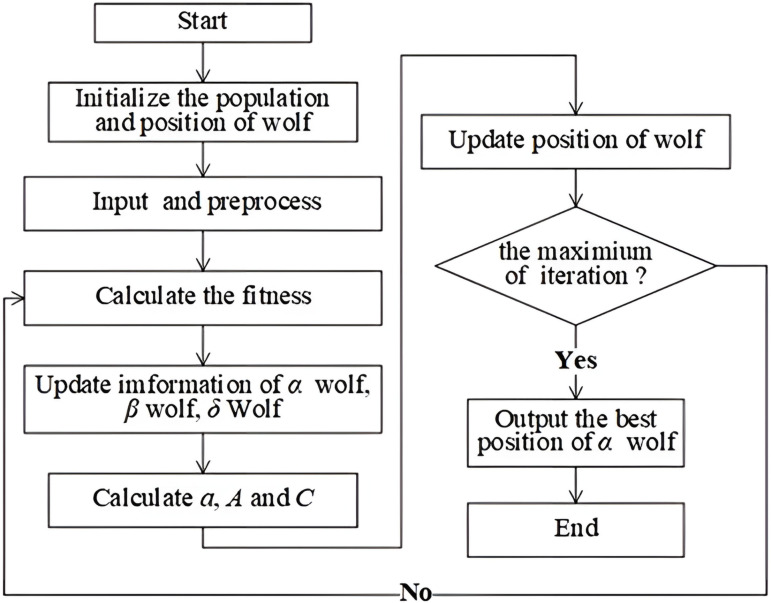
Grey wolf optimizer schematic diagram.

The GWO approach replicates the pack structure and predatory tactics of wild gray wolves. In a pack of gray wolves, four specific hierarchical levels exist: alpha, beta, delta, and omega, as illustrated in Fig 1. These rankings are essential for preserving structure and unity among the members of the pack.

Every individual wolf depicted in Fig 1 fulfills a unique function within its group, wherein the wolves are recognized as the superior alphas, exemplifying the prime, runner-up, and tertiary positions respectively.

Grey wolves’ method of pursuing prey encompasses a trio of critical phases: the initial search for a target, the strategic encirclement of the intended quarry, followed by an enduring chase until the prey ceases motion, culminating with its eventual assault. The progression of these occurrences is depicted as shown in formula (1).


$$\overset{\to }{D}=|\overset{\to }{C}{\overset{\to }{X}}_{p}(t)-\overset{\to }{X}(t)|$$ (1)

vectors ${\overset{\to }{X}}_{p}$ and $\overset{\to }{X}$ reflect the positions of the prey and the gray wolves, respectively, and they not only denote distances but also indicate the ongoing iteration count. Modify the position of the wolves in the subsequent update according to the methodology outlined in equation (2):


$$\overset{\to }{X}(t+1)={\overset{\to }{X}}_{p}(t)-\overset{\to }{A}\overset{\to }{D}$$ (2)

where $\overset{\to }{C}$ and $\overset{\to }{A}$ in (1) and (2) are coefficient vectors, calculated as shown in formula (3):


$$\overset{\to }{A}=2\overset{\to }{a}{\overset{\to }{r}}_{1}-\overset{\to }{a},\overset{\to }{C}=2{\overset{\to }{r}}_{2}$$ (3)

${\overset{\to }{r}}_{1}$ and ${\overset{\to }{r}}_{2}$, which are random numbers between 0 and 1, are involved in the process. The coefficient $\overset{\to }{a}$ undergoes a linear diminution from 2 down to 0 throughout the iteration process, a relationship that is formulated in equation (4).


$$\overset{\to }{a}=2(1-t/T_{\max})$$ (4)

Here, $T_{\max}$ represents the maximum number of iterations.

In the optimization problem’s search domain, the optimal answer (regarding the prey’s position) remains a mystery. Therefore, during the emulation of the gray wolf predation strategy, it is presumed that α, β, and δ wolves have advanced insight into the probable location of the prey. Leveraging the coordinates of these leading wolves, one can infer the prey’s vicinity, which allows the rest of the gray wolf pack to modify their own locations in response. This iterative process allows the wolves to converge towards the prey, as depicted in Fig 1.

Equation (5) depicts the calculation for the vector representing the spatial separation among the trio of wolves, α, β, and δ, throughout their pursuit of the intended quarry.


$$\left\{ \right.\;\begin{array}{c} {\overset{\to }{D}}_{\alpha }=|{\overset{\to }{C}}_{1}{\overset{\to }{X}}_{\alpha }-\overset{\to }{X}|\\ {\overset{\to }{D}}_{\beta }=|{\overset{\to }{C}}_{2}{\overset{\to }{X}}_{\beta }-\overset{\to }{X}|\\ {\overset{\to }{D}}_{\delta }=|{\overset{\to }{C}}_{3}{\overset{\to }{X}}_{\delta }-\overset{\to }{X}| \end{array}$$ (5)

Here, ${\overset{\to }{D}}_{\alpha }$ represents the distance from δwolf to other members of the wolfpack, while ${\overset{\to }{D}}_{\beta }$ and ${\overset{\to }{D}}_{\delta }$ represent the distances from βand δwolf to other members, respectively. Corresponding to this, ${\overset{\to }{X}}_{\alpha }$ and ${\overset{\to }{X}}_{\beta }$, ${\overset{\to }{X}}_{\delta }$ represent the current position vectors of the α, βand δ wolf, as well as the current position vector $\overset{\to }{X}$ of the specified gray wolf being considered. In addition, and ${\overset{\to }{C}}_{1}$ and ${\overset{\to }{C}}_{2}$, ${\overset{\to }{C}}_{3}$ are vectors consisting of random coefficients.

The formula for updating the wolf positions ω is shown as formula (6):


$$\begin{array}{c} \left\{ \right.\;\begin{array}{c} {\overset{\to }{X}}_{1}={\overset{\to }{X}}_{\alpha }-{\overset{\to }{A}}_{1}{\overset{\to }{D}}_{\alpha }\\ {\overset{\to }{X}}_{2}={\overset{\to }{X}}_{\beta }-{\overset{\to }{A}}_{2}{\overset{\to }{D}}_{\beta }\\ {\overset{\to }{X}}_{3}={\overset{\to }{X}}_{\delta }-{\overset{\to }{A}}_{3}{\overset{\to }{D}}_{\delta } \end{array}\\ \overset{\to }{X}(t+1)=\frac{{\overset{\to }{X}}_{1}+{\overset{\to }{X}}_{2}+{\overset{\to }{X}}_{3}}{3} \end{array}\;$$ (6)

Here, ${\overset{\to }{X}}_{1}$ and ${\overset{\to }{X}}_{2}$, ${\overset{\to }{X}}_{3}$ respectively represent the current positions of the three wolves. ${\overset{\to }{C}}_{1}$ and ${\overset{\to }{C}}_{2}$, ${\overset{\to }{C}}_{3}$ and are vectors consisting of random coefficients.

### 2.2. Multilayer Perceptron

**Fig 2:**
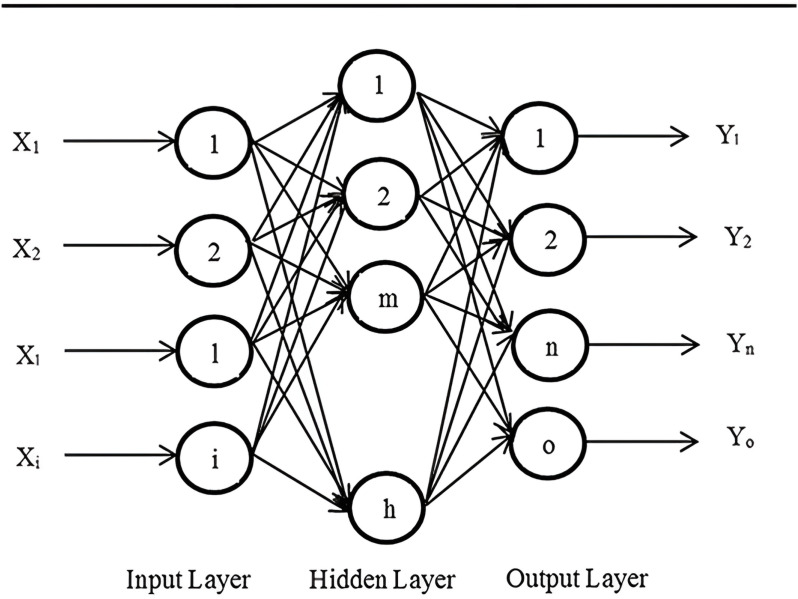
BP schematic diagram.

A widely employed architecture for categorizing patterns and estimating continuous functions [23] is the feedforward neural network (FNN). Characterized by its sequentially layered arrangement of neurons. The Backpropagation (BP) algorithm typically consists of at least two layers. Within the Backpropagation architecture, each successive layer (labeled as i + 1) functions as the recipient of the preceding layer’s (denoted as i) output, whilst neurons situated within an identical layer do not engage in direct interchange of information. The framework’s inception and culmination points are designated as the input and output layers, in that order (see Fig 2).

The quantity of units in the initial layer is equivalent to the quantity of attributes in the input vector, while the quantity of units in the final layer denotes the quantity of unique output categories [23]. Here, ‘i’ stands for the input nodes, ‘h’ denotes the hidden nodes, and ‘o’ indicates the output nodes. The output value of the nth node can be computed utilizing the formula as follows:


$$\begin{array}{c} \begin{array}{cc} Y_{n} & =f(\sum_{m=1}^{h}(w_{nm}f(\sum_{l=1}^{i}v_{ml}X_{l}+\theta_{vm})+\theta_{wm})) \end{array} \end{array}$$

n=1,…,o (7)

Within this framework, the representation for the output from the node n in the final layer is denoted by $Y_{n}$. The initial layer’s node 1 receives an input designated $X_{l}$, and the weight of the link from node m in the intermediary layer to node n in the terminal layer is expressed as $w_{nm}$. Furthermore, the term $v_{ml}$ describes the link weight from node l in the entrance layer to node m in the intermediate layer. The placeholders $\theta_{vm}$ and $\theta_{wm}$ signify the bias or threshold associated with the transfer function f, functioning from the mth hidden layer neuron to the nth output layer neuron.

The hidden and output layers utilize the sigmoid function as their activation mechanism.


$$f(x)=Sigmoid(x)=\frac{1}{\left(1+exp(-x)\right)}$$ (8)

Each neuron contributes its synaptic weights and bias values to the overall network’s weight matrix. Refining the values of these weights and biases to pinpoint the best parameters is referred to as the training phase of the neural network [24].

## 3. The suggested method

### 3.1. Introduction to LGWO

In order to address the search deviation issue often faced in the traditional Grey Wolf Optimization (GWO) algorithm, several enhancements have been proposed. The manuscript presents an innovative method for training neural networks that amalgamates Levy flight strategies, GWO, and the Backpropagation (BP) technique. This synergistic approach capitalizes on GWO’s proficiency in extensive exploration and the BP algorithm’s adeptness in specific refinement by utilizing Levy flight’s advantageous properties to augment GWO’s capacity for comprehensive searching, thus averting the potential pitfall of convergence to suboptimal solutions.Incorporating the Backpropagation algorithm substantially improves the GWO algorithm’s capacity for localized optimization.

### 3.2. Encoding process

**Fig 3:**
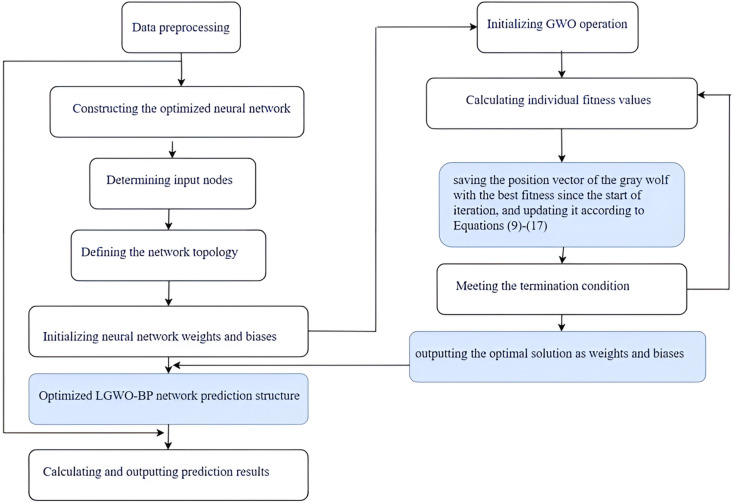
LGWO-BP research flowchart.


Fig 3 show the LGWO-BP Research Flowchart. The manuscript presents an innovative method wherein the weight factor is defined by a function within the grey wolf optimization algorithm, described as follows:

$$O=((T_{\max}-2)/T_{\max})\cdot {\left(w_{s}-(w_{s}-w_{e})\cdot \arctan(a+b\cdot t)\cdot \frac{2}{\pi }\right)}^{2}$$ (9)

The iteration count is denoted by t, with the maximum allowable iterations being $T_{\max}$, while $w_{s}$ holds a value of 1.5, $w_{e}$ is set at 0.1, and the constants a and b are assigned 1 and 0.01, respectively. This algorithm simulates the competition and cooperation mechanisms among cuckoo populations [26].Drawing on the principles of the cuckoo algorithm, the study incorporates Levy flights [27]. Levy flights encompass the random selection of direction and step size within the exploration space, simulating the unpredictable movement of birds to improve the algorithm’s ability to investigate areas around local peaks [28].If the grey wolf algorithm fails to converge on the optimal solution following a specified number of iterations, the plan involves adopting a different search strategy based on Levy flight to avoid getting stuck in local minima [29].A Levy flight represents a random walk where the step sizes comply with Levy’s statistical distribution [30]. This distribution is based on a simple weight-rule formula $L(s)\sim |s{|}^{-1-\beta }$. where0<β≤2 is an index. The following presents a straightforward formula for the Levy distribution:


$$L(s,\gamma ,\mu )=\left\{ \right.\;\begin{array}{cc} \sqrt{\frac{\gamma }{2\pi }}\exp[-\frac{\gamma }{2(s-\mu )}]\frac{1}{(s-\mu {)}^{\frac{3}{2}}} & \;if\;0<\mu <\infty \\ 0 & \;if\;s\leq 0 \end{array}$$ (10)

Within this group, μ signifies the location or movement parameter, while γ represents the parameter governing the spread’s scale, with s denoting the collection of samples within this spread. The technique introduced in our research initiates by creating a randomized assembly of wolves, subsequently determining the cost associated with each individual wolf, followed by identifying the wolves. The process of surrounding, hunting, and attacking prey is repeated until the algorithm results show no significant improvement after a certain number of iterations.Presently, the search will persist with Levy flights maneuvering the wolves across the newly designated search area.


$$S=0.01*\frac{u}{v^{1/\beta }}*(X-X_{\alpha })$$ (11)

where u and v denote randomly selected numerical values from a normal distribution.

Greedy strategy is a problem-solving method that involves choosing the best possible option at each step, considering only the immediate benefit without considering future consequences [31].The approach of using a greedy algorithm is frequently employed in optimization challenges, including those involving minimum spanning trees and finding the shortest routes. Employing this technique involves utilizing a greedy algorithm to evaluate the fitness values and retaining the superior fitness value as the optimal choice.

where L is a Levy random number.


$$x_{new}=x_{best}+O\cdot L\cdot \left(x_{best}-x_{wort}\right)$$ (12)

where L is a Levy random number.

The technique known as Opposition-based Learning (OBL) improves the performance and productivity of computational learning processes through the utilization of antithetical principles. This approach fundamentally aims to augment the variability and competitive aspect within the training phase by inserting counterpart examples that act as the antithesis to the existing instances. In OBL, each positive sample has an opposing negative sample as a control. These sample pairs are designed to be opposite in some features or attributes. By contrasting positive and opposing negative samples, OBL can increase robustness and improve the generalization ability of classifiers [32]. This study also uses opposition-based learning produce $x_{new}$.


$$x_{new}=ub+lb-L\cdot x_{best}$$ (13)

$$x^{1}(t+1)=x(t)+L\cdot (x_{best}-x(t))$$ (14)

$$x^{2}(t+1)=O\cdot x(t)$$ (15)

Adhering to a greedy approach, the fitness scores of both $x_{new}$ and $x_{best}$ are evaluated anew, with the superior one being preserved as $x_{best}$.

Inspired by the Harris Hawks Optimization algorithm, in this study, each individual generates a random number q, and when q is less than the mutation probability pp, the following mutation operations are performed:


$$x^{3}(t+1)=x_{r}(t)-rand\cdot |x_{r}(t)-2\cdot rand\cdot x(t)|$$ (16)

$$x^{4}(t+1)=(x_{best}-x_{m})-rand\cdot ((ub-lb)\cdot rand+lb)$$ (17)

Comparing the fitness values of $x^{3}(t+1)$, $x^{4}(t+1)$ and x(t+1), using a greedy strategy, the best one is retained as x(t+1).

The comprehensive structure of the proposed methodology unfolds as follows:

**Phase One: Setup** – An array of wolves, each symbolizing a viable solution, is randomly assembled, followed by the calculation of each wolf’s cost by assessing it via the objective function.
**Step 2: Identification** – The α, β, and δ wolves, corresponding to the best, second-best, and third-best solutions, respectively, are pinpointed and designated.
**Step 3: Iteration** – The sequence of encircling, pursuing, and attacking the prey (aimed at optimizing the objective function) is iterated. This process entails updating the wolves’ positions relative to the α, β, and δ wolves.
**Step 4: Levy Flight** – If no substantial improvement is noted after a predefined number of iterations, Levy flights are integrated. This strategy entails randomly adjusting the positions of certain wolves using the Levy distribution to facilitate evasion from local minima.
**Step 5: Opposition-based Learning (OBL)** – The efficacy and efficiency of algorithmic learning are augmented by incorporating the principle of contrariety through OBL.
**Step 6: Fitness Evaluation** – Referencing Equations (14) and (15), the fitness scores of both Xnew and Xbest are reassessed, with the superior one being retained as Xbest.
**Step 7: Mutation Inspired by Harris Hawks Optimization** – Each individual generates a random number q.Should q fall below the mutation probability, pp, one should implement Equations (16) and (17).
**Step 8: Greedy Algorithm Evaluation** – A greedy algorithm is utilized to assess fitness values, retaining the highest as the optimal choice.
**Phase 9** Implementation of the LGWO Technique – The LGWO technique is utilized to fine-tune the synaptic weights and offsets across the layers of the BP neural network.
**Step 10: BP Algorithm Search** – The BP algorithm is applied to explore the solutions’ proximity, refining the optimization process.

The GWO is renowned for its robust global search prowess, adept at exploring vast solution spaces. Conversely, while the Back Propagation (BP) algorithm may encounter slower convergence rates as it nears optimality, it compensates with exceptional local search capabilities, albeit with limitations in its global search reach. The output accuracy of the BP model is intimately tied to the weights and biases within its network structure, where meticulous design of these parameters can minimize network errors and enhance prediction precision.

At the outset, the BP network’s parameters, including weights and biases, are set to random values and are later precisely adjusted using the technique of gradient descent. However, this iterative adjustment process can be marred by slow convergence and the risk of getting trapped in local optima.

Welcome the Leader Grey Wolf Optimizer, a strategic problem-solving method that replicates the instinctive pack hierarchy and predatory tactics of grey wolves. It boasts rapid convergence, adaptability, and robust generalization capabilities, particularly excelling in its global search strategy.This feature enhances the combined model’s predictive precision by countering the backpropagation algorithm’s propensity to become ensnared in local troughs.

Pseudocode for LGWO-BP Algorithm Implementation

Algorithm Pseudo-code of the LGWO-BP

**Initialize:** Wolves randomly generated, costs calculated via function.

**Identification:** Best, 2nd-best, 3rd-best wolves are identified and named.

**Iteration:** Encircling, pursuing, attacking prey is iterated, updating positions.

**Levy Flight**: If no significant improvement is observed after the predetermined iterations, Levy flights are employed to adjust positions.

**Opposition-based Learning (OBL):** OBL enhances algorithmic learning’s efficacy and efficiency.

**Fitness Evaluation:** Using Eqs. (14) and (15), reassess Xnew and Xbest, keeping the better as Xbest.

Mutation Inspired by Harris Hawks Optimization: If q < pp, apply Eqs. (16) and (17).

**Greedy Algorithm Evaluation:** A greedy algorithm retains the highest fitness as optimal.

**Implementation of the LGWO Technique:** LGWO fine-tunes synaptic weights and offsets in BP neural network layers.

**BP Algorithm Search:** The BP algorithm is applied to explore the solutions’ proximity, refining the optimization process.

In this study, we adopted an automated method based on the Grey Wolf Optimizer (GWO) algorithm to fine-tune the weights and biases of a neural network. The following provides detailed information on the specifics of parameter tuning and model confi Recent works in the field of metaheuristic optimization have highlighted the growing interest in applying such techniques to medical applications, particularly in the areas of disease diagnosis, prognosis, and treatment planning. For instance, several studies have explored the use of genetic algorithms, particle swarm optimization, and other metaheuristic approaches to optimize various aspects of medical decision-making processes [21–24]. These works have demonstrated the potential of metaheuristics in improving the accuracy and efficiency of medical models, aligning with the objectives of our study.

Network Structure Settings:

- Hidden Layer Dimension (num_hidden): Set to 6, which is an empirically based choice but can be adjusted according to the specific needs of the problem to achieve optimal network performance.
- The initial values of weights and biases are generated randomly to provide diverse starting points for the optimization process. These parameters will be adjusted based on the values of the fitness function during the subsequent optimization process.

Grey Wolf Optimizer Parameters:

- Number of Search Agents (SearchAgents_no): Set to 5 based on experimental trials on a small dataset to ensure sufficient diversity in the search space.
- Maximum Number of Iterations (Max_iteration): Set to 30 through experimental results on the same dataset to find a superior solution within limited computational resources.
- Parameter Value Bounds (lb and ub): Set to −1 and 1, respectively, to limit the search space and ensure the stability and effectiveness of the optimization process.
- Fitness Function (fitcal):
- The fitness function is used to evaluate the quality of each candidate solution. Its specific implementation involves the training error of the neural network, with the goal of minimizing this error to find the optimal network parameter configuration.

During each iteration, the positions of the Alpha, Beta, and Delta wolves are updated by calculating the fitness function values, guiding the wolf pack towards a better solution set. A linear weight decrease strategy and a random exploration strategy are employed to balance global search and local exploitation, ensuring that the optimization process possesses both global and local fine-search capabilities. The updated parameters are used to adjust the weights and biases of the neural network, gradually optimizing its performance. The neural network is then configured with the optimized parameters and trained. After training, the model’s performance on both the training and test sets is evaluated through simulation predictions to verify the effectiveness of the optimization method. Through detailed parameter tuning and model configuration, we ensure the optimal performance of the neural network for specific problems.

Fundamentally, this blended approach enhances the effectiveness of the search for the best solution, hastens the convergence process, and guarantees that the Backpropagation algorithm achieves the highest level of accuracy with the high-potential selections identified by LGWO.

## 4. Simulation experiment and result analysis

### 4.1. Experimental settings

In order to assess the accuracy and efficiency of our advanced LGWO-BP predictive framework for forecasting cancer outcomes, we structured multiple simulated trials utilizing MATLAB. The purpose of these trials extends beyond evaluating the enhanced capabilities of the Leveraged Grey Wolf Optimizer (LGWO).

These tests were conducted on a system equipped with an Intel Core i7 processor, which operates at a frequency of 3.20GHz, includes 4GB of memory, and utilizes a 64-bit version of the Windows 10 OS. To rigorously evaluate the LGWO algorithm’s capabilities, we employed a comprehensive set of 18 established benchmark functions [33,34]. These functions were carefully selected to mimic the optimization challenges encountered when training a neural network for cancer prognosis prediction.

Single-modal benchmark functions serve as a baseline to assess the LGWO algorithm’s global search ability, which is crucial for identifying promising regions in the parameter space for the BP network. At the same time, diverse composite benchmarks assess the capacity of the algorithm to overcome local maxima and carry out productive proximate examinations, similar to the adjustment of weights and biases in neural networks for enhanced forecast precision.

Furthermore, we evaluated the LGWO algorithm across various dimensional settings, from smaller to larger scales, to demonstrate its adaptability and robustness in handling optimization problems of different complexities. This adaptability is essential when scaling the optimization process to larger neural networks and more complex cancer prognosis prediction tasks.

By leveraging these benchmark functions, we aim to provide empirical evidence that the LGWO algorithm can efficiently optimize the parameters of the neural network, thereby improving its predictive performance for cancer prognosis. In the end, this research advances the creation of computational models that are both more precise and trustworthy for forecasting the prognosis of cancer.

The details of the single-peaked benchmark functions are shown in Table 1, listing the function expressions, dimensions, search ranges, and optimal solutions.

**Table 1:** Single-peaked reference functions.

Function	Dim	Search Ranges	Optimal Solutions
$f_{1}(x)=\sum_{i=1}^{n}x_{i}^{2}$	30	[−100, 100]	0
$f_{2}(x)=\sum_{i=1}^{n}|x_{i}|+\prod_{i=1}^{n}|x_{i}|$	30	[− 10, 10]	0
$f_{3}(x)=\sum_{i=1}^{n}{\left(\sum_{j=1}^{i}x_{j}\right)}^{2}$	30	[−100, 100]	0
$f_{4}(x)=m_{i}\left\{|x_{i}|,1⩽i⩽n\right\}$	30	[−100, 100]	0
$f_{5}(x)=\sum_{i=1}^{n-1}[100{\left(x_{i+1}-x_{i}^{2}\right)}^{2}+{\left(x_{i}-1\right)}^{2}]$	30	[−30, 30]	0
$f_{6}(x)=\sum_{i=1}^{n}ix_{i}^{4}+random[0,1)$	30	[−1.28, 1.28]	0

Table 2 presents the capabilities of the multi-faceted standard examination.

**Table 2:** Multimodal reference functions.

Function	Dim	Search Ranges	Optimal Solutions
$f_{7}(x)=\sum_{i=1}^{n}-x_{i}\sin\left(\sqrt{\left|x_{i}\right|}\right)$	30	[−500, 500]	−12,569.5
$f_{8}(x)=\sum_{i=1}^{n}[x_{i}^{2}-10\cos(2\pi x_{i})+10]$	30	[−5.12, 5.12]	0
$f_{9}(x)=-20\exp(-0.2\sqrt{\frac{1}{n}\sum_{i=1}^{n}x_{i}^{2})}-\exp(\frac{1}{n}\sum_{i=1}^{n}\cos(2\pi x_{i}))+20+e$	30	[−32, 32]	0
$f_{10}(x)=\frac{1}{4000}\sum_{i=1}^{n}x_{i}^{2}-\prod_{i=1}^{n}cos(\frac{x_{i}}{\sqrt{i}})+1$	30	[−600, 600]	0
$\begin{array}{c} \begin{array}{c} f_{11}(x)=0.1\{\sin^{2}(3\pi \;x_{1})+\sum_{i=1}^{n-1}{\left(x_{i}-1\right)}^{2}[1+\sin^{2}(3\pi \;x_{i+1})]\\ +{\left(x_{n}-1\right)}^{2}[1+\sin^{2}(2\pi \;x_{n}]+\sum_{i=1}^{n}u(x_{i},5,100,4) \end{array} \end{array}$	30	[−50, 50]	0

Table 3 presents the reference functions for fixed-dimensional multimodality.

**Table 3:** Fixed-dimensional multimodal reference functions.

Function	Dim	Search Ranges	Optimal Solutions
$f_{12}(x)={\left[\frac{1}{500}+\sum_{j=1}^{25}\frac{1}{j+\sum_{i=1}^{2}{\left(x_{i}-a_{ij}\right)}^{6}}\right]}^{-1}$	2	[−65, 65]	1
$f_{\begin{array}{c} 13 \end{array}}(x)=\sum_{i=1}^{11}{\left[a_{i}-\frac{x_{1}\left(b_{i}^{2}+b_{i}x_{2}\right)}{b_{i}^{2}+b_{i}x_{3}+x_{4}}\right]}^{2}$	4	[−5, 55]	0.00E + 0003
$f_{14}(x)=4x_{1}^{2}-2.1x_{1}^{4}+\frac{1}{3}x_{1}^{6}+x_{1}x_{2}-4x_{2}^{2}+4x_{2}^{4}$	2	[−5, 55]	−1.03162
$f_{15}(x)={\left(x_{2}-\frac{5.1}{4\pi^{2}}x_{1}^{2}+\frac{5}{\pi }x_{1}-6\right)}^{2}+10(1-\frac{1}{8\pi })cosx_{1}+10$	2	[−5, 55]	0.398
$\begin{array}{c} f_{16}(x)=[1+{\left(x_{1}+x_{2}+1\right)}^{2}(19-14x_{1}+3x_{1}^{2}-14x_{2}\;\;\\ \;\;+6x_{1}x_{2}+3x_{2}^{2})]\times [30+{\left(2x_{1}-3x_{2}\right)}^{2}(18-32x_{1}\;\;\\ \;\;+12x_{1}^{2}+48x_{2}-36x_{1}x_{2}+27x_{2}^{2})] \end{array}$	2	[−2, 22]	3
$f_{17}(x)=-\sum_{i=1}^{4}c_{i}\exp[-\sum_{j=1}^{3}a_{ij}{\left(x_{j}-p_{ij}\right)}^{2}]$	3	[0, 1]	−3.86
$f_{18}(x)=-\sum_{i=1}^{4}c_{i}\exp[-\sum_{j=1}^{6}a_{ij}{\left(x_{j}-p_{ij}\right)}^{2}]$	6	[0, 1]	−3.32

### 4.2. Assessing the Efficacy of LGWO: A comparative analysis with alternative optimization methods

This study assesses the efficacy of LGWO by benchmarking it against an array of algorithms including TSA [35], ROA [36], DBO [37], RUN [38], GJO [39], LCA [40], PSA [41], BKA [42], as well as the conventional GWO intelligent algorithm and the suggested LGWO approach. Evaluation of each algorithm’s capabilities is conducted through the analysis of the best, mean, and variability in results derived from their application to 18 distinct test functions. The test statistical data are as follows.

Table 4 unequivocally demonstrates the LGWO algorithm’s outstanding performance in single-peak benchmark functions. Particularly, regarding functions f1 through f4, the LGWO algorithm consistently attains the theoretical maximum, with the standard deviation for these functions being nil, which signifies that its solution quality significantly exceeds that of competing algorithms. Across the remaining tasks, LGWO exceeds the performance of alternative algorithms, while its standard deviation regarding solution accuracy also indicates the algorithm’s reliable consistency. Single-peak benchmark functions are frequently employed to assess the global search efficiency of algorithms. The analysis presented demonstrates that the LGWO algorithm exhibits robust global search capabilities.

**Table 4:** Outcomes of unimodal benchmarking assessments.

Functions		f1			f2	
Results	Best	Mean	Std	Best	Mean	Std
BKA	3.63E-134	1.18E-110	5.42E-110	3.02E-66	1.49E-60	5.70E-60
DBO	1.52E-199	7.20E-138	3.94E-137	3.49E-106	8.63E-68	4.73E-67
GJO	2.10E-81	6.14E-78	1.98E-77	3.17E-47	9.88E-46	1.74E-45
GWO	5.55E-42	1.12E-40	2.42E-40	6.01E-25	4.40E-24	4.03E-24
LCA	4.78E-04	3.98E-02	4.44E-02	1.72E-02	1.17E-01	7.30E-02
PSA	7.79E-07	4.15E-05	8.57E-05	9.58E-04	9.43E-03	1.66E-02
ROA	3.93E-33	5.48E-15	2.61E-14	6.42E-15	1.39E-08	6.29E-08
RUN	1.24E-260	1.96E-239	0.00E + 00	1.29E-141	8.71E-132	4.72E-131
TSA	6.75E-32	2.32E-29	8.66E-29	9.49E-20	1.87E-18	3.19E-18
LGWO	**0.00E + 00**	**0.00E + 00**	**0.00E + 00**	**0.00E + 00**	**0.00E + 00**	**0.00E + 00**
Functions		f3		f4		
Results	Best	Mean	Std	Best	Mean	Std
BKA	3.88E-130	2.81E-102	1.54E-101	8.54E-66	1.15E-56	6.18E-56
DBO	5.59E-179	3.37E-86	1.84E-85	6.02E-106	3.26E-66	1.77E-65
GJO	1.69E-31	9.67E-24	5.25E-23	5.56E-25	7.93E-22	2.22E-21
GWO	1.70E-13	7.03E-10	2.00E-09	3.10E-11	3.84E-10	5.19E-10
LCA	5.93E-02	1.54E + 01	2.38E + 01	5.89E-03	4.20E-02	3.83E-02
PSA	2.14E + 02	4.77E + 02	1.86E + 02	1.64E + 00	5.86E + 00	2.97E + 00
ROA	1.12E-29	4.54E-14	2.35E-13	5.59E-20	1.32E-09	6.42E-09
RUN	3.63E-225	2.53E-204	0.00E + 00	3.39E-123	2.31E-112	8.24E-112
TSA	1.53E-14	1.52E-08	5.71E-08	3.22E-04	3.10E-02	4.54E-02
LGWO	**0.00E + 00**	**0.00E + 00**	**0.00E + 00**	**0.00E + 00**	**0.00E + 00**	**0.00E + 00**
Functions		f5			f6	
Results	Best	Mean	Std	Best	Mean	Std
BKA	2.49E + 01	2.72E + 01	1.01	2.11E-06	2.11E-06	1.35E-04
DBO	2.44E + 01	2.47E + 01	1.50E-01	7.35E-05	1.55E-03	1.37E-03
GJO	2.62E + 01	2.74E + 01	7.36E-01	2.90E-05	3.07E-04	2.79E-04
GWO	2.55E + 01	2.68E + 01	6.87E-01	2.80E-04	1.16E-03	9.94E-04
LCA	3.79E-04	4.18E-01	6.35E-01	1.79E-04	6.89E-02	1.06E-01
PSA	3.18	6.77E + 01	4.59E + 01	1.22E-01	2.85E-01	1.21E-01
ROA	1.08E-05	5.52E-02	1.24E-01	8.46E-06	1.78E-04	1.60E-04
RUN	2.06E + 01	2.30E + 01	1.27	1.30E-05	1.53E-04	1.11E-04
TSA	2.60E + 01	2.83E + 01	9.62E-01	2.50E-03	7.14E-03	2.75E-03
LGWO	7.95E-04	7.56E-03	4.47E-03	1.58E-07	1.78E-05	1.70E-05

The results presented in Table 5, Table 6 indicate that LGWO exhibits strong solutionodal benchmark functions.Particularly, with regards to the functions f8 and f10, LGWO attains the optimal theoretical values in terms of best, mean, and variance measures, boasting a variance of zero, thus markedly outperforming competing algorithms. This analysis clearly illustrates the robust solution performance of LGWO in tackling multimodal benchmark functions.

**Table 5:** Test results of multimodal benchmark functions.

Functions		f7			f8	
Results	Best	Mean	Std	Best	Mean	Std
BKA	−1.12E + 04	−9.33E + 03	1.22E + 03	0.00E + 00	0.00E + 00	0.00E + 00
DBO	−1.16E + 04	−9.13E + 03	1.26E + 03	0.00E + 00	1.03E + 00	4.09E + 00
GJO	−6.58E + 03	−4.57E + 03	1.21E + 03	0.00E + 00	0.00E + 00	0.00E + 00
GWO	−7.33E + 03	−5.98E + 03	9.37E + 02	0.00E + 00	4.17E-01	1.37E + 00
LCA	−1.26E + 04	−1.11E + 04	2.88E + 03	1.02E-03	3.59E-02	5.40E-02
PSA	−1.10E + 04	−1.02E + 04	4.59E + 02	1.89E + 01	4.44E + 01	1.39E + 01
ROA	−1.26E + 04	−1.26E + 04	8.63E-04	0.00E + 00	7.58E-15	4.15E-14
RUN	−9.15E + 03	−8.29E + 03	4.45E + 02	0.00E + 00	0.00E + 00	0.00E + 00
TSA	−7.41E + 03	−6.36E + 03	6.77E + 02	8.52E + 01	1.64E + 02	3.66E + 01
LGWO	−1.01E + 176	−3.35E + 174	Inf	**0.00E + 00**	**0.00E + 00**	**0.00E + 00**
Functions		f9			f10	
Results	Best	Mean	Std	Best	Mean	Std
BKA	4.44E-16	4.44E-16	0.00E + 00	0.00E + 00	0.00E + 00	0.00E + 00
DBO	4.44E-16	4.44E-16	0.00E + 00	0.00E + 00	5.21E-04	2.85E-03
GJO	4.00E-15	5.54E-15	1.79E-15	0.00E + 00	0.00E + 00	0.00E + 00
GWO	1.47E-14	2.79E-14	4.37E-15	0.00E + 00	2.44E-03	5.84E-03
LCA	7.78E-03	5.24E-02	4.27E-02	5.65E-03	1.74E-01	2.16E-01
PSA	3.99E-04	5.25E-01	6.56E-01	3.83E-06	2.88E-02	2.96E-02
ROA	4.44E-16	2.73E-10	8.08E-10	0.00E + 00	8.29E-16	3.99E-15
RUN	4.44E-16	4.44E-16	0.00E + 00	0.00E + 00	0.00E + 00	0.00E + 00
TSA	1.47E-14	1.84E + 00	1.66E + 00	0.00E + 00	1.19E-02	1.25E-02
LGWO	4.44E-16	4.44E-16	**0.00E + 00**	**0.00E + 00**	**0.00E + 00**	**0.00E + 00**

**Table 6:** Test results of multimodal benchmark functions.

Functions		f11	
Results	Best	Mean	Std
BKA	5.22E-01	1.58E + 00	5.24E-01
DBO	3.80E-11	1.44E-01	1.55E-01
GJO	6.79E-01	1.40E + 00	3.07E-01
GWO	4.30E-05	2.67E-01	1.71E-01
LCA	2.10E-06	4.92E-03	6.25E-03
PSA	4.72E-07	6.06E-02	2.91E-01
ROA	2.08E-07	1.26E-03	2.87E-03
RUN	1.20E-09	5.03E-03	7.27E-03
TSA	1.86E + 00	2.96E + 00	5.61E-01
LGWO	3.00E-07	6.99E-06	5.25E-06

The effectiveness of approaches on mixed-mode benchmark tasks often reflects the algorithm’s local optimization skills and its capacity to steer clear of becoming ensnared in suboptimal local peaks. Based on the test outcomes, LGWO demonstrates proficient local search capabilities and excels in avoiding local optimal solutions.

Based on the data provided in Table 7, Table 8, the LGWO algorithm showcases impressive solution performance when addressing fixed-dimensional multimodal benchmark functions.For every benchmark function tested, the top results secured by LGWO are nearly equivalent to the calculated ideal value, featuring numerous cases in which the algorithm attains or indeed exceeds the envisioned best-case scenario. Notably, for function f13, LGWO demonstrates the best standard deviation among the results.In the realm of multimodal benchmark functions with a set dimensional count, the superior search outcomes demonstrated by LGWO emphatically validate its proficiency in executing comprehensive global searches and avoiding suboptimal local solutions in static, low-dimensional contexts. This fully utilizes the results of Levy flights, demonstrating the effectiveness of LGWO.

**Table 7:** Table 7 displays the results of evaluations performed on fixed-dimension multimodal benchmark functions.

Functions		f12			f13	
Results	Best	Mean	Std	Best	Mean	Std
BKA	9.98E-01	1.03E + 00	1.81E-01	3.07E-04	1.11E-03	4.06E-03
DBO	9.98E-01	1.23E + 00	9.59E-01	3.07E-04	7.31E-04	3.47E-04
GJO	9.98E-01	4.07E + 00	3.86E + 00	3.08E-04	1.04E-03	3.65E-03
GWO	9.98E-01	3.55E + 00	3.76E + 00	3.07E-04	3.11E-03	6.89E-03
LCA	9.98E-01	1.19E + 01	1.17E + 01	3.56E-04	1.78E-02	3.72E-02
PSA	9.98E-01	9.98E-01	2.33E-16	3.07E-04	3.86E-03	7.51E-03
ROA	9.98E-01	1.06E + 00	3.62E-01	3.09E-04	4.09E-04	1.19E-04
RUN	9.98E-01	2.41E + 00	2.46E + 00	3.07E-04	4.91E-04	3.73E-04
TSA	9.98E-01	7.82E + 00	5.47E + 00	3.08E-04	1.15E-02	2.11E-02
LGWO	9.98E-01	9.98E-01	3.84E-14	3.07E-04	3.57E-04	1.78E-04

**Table 8:** Results from evaluating fixed-dimension multimodal benchmark tests.

Functions		f14			f15	
Results	Best	Mean	Std	Best	Mean	Std
BKA	−1.03	−1.03	6.25E-16	3.98E-01	3.98E-01	0.00E + 00
DBO	−1.03	−1.03	6.58E-16	3.98E-01	3.98E-01	0.00E + 00
GJO	−1.03	−1.03	8.32E-08	3.98E-01	3.98E-01	2.21E-06
GWO	−1.03	−1.03	3.50E-09	3.98E-01	3.98E-01	9.76E-08
LCA	−1.03	−8.47E-01	2.23E-01	4.01E-01	5.32E-01	1.44E-01
PSA	−1.03	−1.03	5.45E-16	3.98E-01	3.98E-01	0.00E + 00
ROA	−1.03	−1.03	1.74E-05	3.98E-01	4.28E-01	1.13E-01
RUN	−1.03	−1.03	1.08E-13	3.98E-01	3.98E-01	2.73E-13
TSA	−1.03	−1.03	1.03E-07	3.98E-01	3.98E-01	9.07E-06
LGWO	−1.03	−1.03	7.40E-12	3.98E-01	3.98E-01	1.06E-10
Functions		f16			f17	
Results	Best	Mean	Std	Best	Mean	Std
BKA	3	3	1.04E-15	−3.86E + 00	−3.86E + 00	2.58E-15
DBO	3	3	1.10E-15	−3.86E + 00	−3.86E + 00	2.40E-03
GJO	3	3	7.92E-07	−3.86E + 00	−3.86E + 00	3.99E-03
GWO	3	3	1.29E-05	−3.86E + 00	−3.86E + 00	2.37E-03
LCA	3.33	1.80E + 01	1.10E + 01	−3.81E + 00	−3.39E + 00	3.28E-01
PSA	3	3	2.42E-15	−3.86E + 00	−3.86E + 00	2.44E-15
ROA	3	3	2.51E-04	−3.86E + 00	−3.81E + 00	7.61E-02
RUN	3	3	1.48E-13	−3.86E + 00	−3.86E + 00	1.50E-09
TSA	3	4.8	6.85	−3.86E + 00	−3.86E + 00	3.60E-05
LGWO	3	3	9.04E-07	−3.86	−3.86	1.58E-03
Functions		f18				
Results	Best	Mean	Std			
BKA	−3.32	−3.3	4.51E-02			
DBO	−3.32	−3.23	8.41E-02			
GJO	−3.32	−3.16	1.26E-01			
GWO	−3.32	−3.26	6.92E-02			
LCA	−2.5	−1.9	4.32E-01			
PSA	−3.32	−3.27	6.03E-02			
ROA	−3.25	−3.01	1.66E-01			
RUN	−3.32	−3.27	6.03E-02			
TSA	−3.32	−3.27	6.97E-02			
LGWO	−3.32	−3.28	6.90E-02			

**Fig 4:**
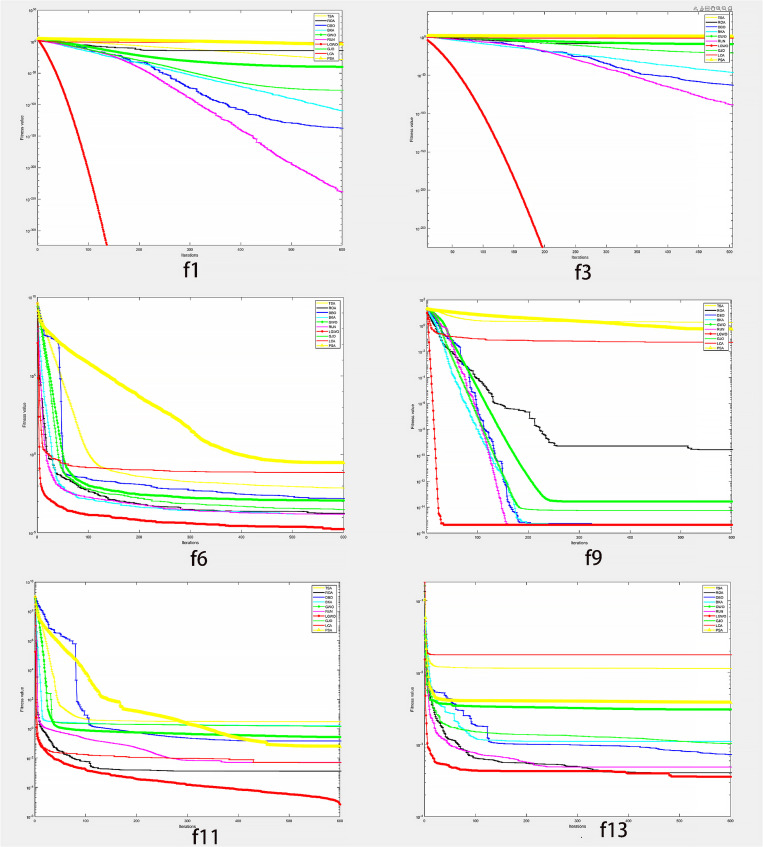
The convergence curves of the test functions.

By analyzing the results of the aforementioned 3 categories of test functions (Tables 4–8), it can be inferred that LGWO holds considerable advantages in terms of accuracy, stability, and scalability across various types of test functions.Fig 4 offers a graphical depiction of LGWO’s capabilities by displaying the convergence trends of the test functions.

Fig 4 illustrates that the LGWO algorithm converges rapidly, particularly when applied to single-peak optimization functions. This algorithm exhibits a markedly swifter rate of convergence relative to its counterparts, displaying enhanced precision through its effective approach in nearing the theoretical optimum or its nearest estimate. Examination of the iteration graph for function f11 unmistakably reveals that while other algorithms tend to converge towards local optimal values, LGWO excels at breaking away from such local optima, underscoring its exceptional capability to evade local optimal solutions. In addition, it fully demonstrates the advantages of Levy flights.For the functions f6, f9, and f13, despite the LGWO algorithm facing similar issues to other methods when it comes to being trapped in suboptimal local solutions, it greatly surpasses these other methods with regards to the precision of the solutions and the rapidity of convergence. Furthermore, the ideal figures achieved through LGWO are the nearest approximation to the theoretical optimum.

In summary, LGWO demonstrates notable advantages in accuracy, stability, scalability, and convergence, providing strong support for the feasibility LGWO algorithm.

This study employs the Wilcoxon rank-sum test for assessing the performance and optimization potential of the LGWO algorithm by investigating if there are significant disparities in the operational results of the LGWO [31]. Comparative outcomes for the LGWO, along with those of TSA [35], ROA [36], DBO [37], RUN [38], GJO [39], LCA [40], PSA [41], BKA [42], and GWO algorithms, are presented in the accompanying table. It is evident from Table 9 that, for the majority of test functions, the p-values associated with LGWO are below the significance level a, signifying notable variances in the computation results when compared to other algorithms.

**Table 9:** The rank sum test p-value.

	f1	f 2	f 3	f 4	f 5	f 6
**LGWO vs. TSA**	1.21E-12	1.21E-12	1.21E-12	1.21E-12	3.02E-11	3.02E-11
**LGWO vs. ROA**	1.21E-12	1.21E-12	1.21E-12	1.21E-12	6.00E-01	5.53E-08
**LGWO vs. DBO**	1.21E-12	1.21E-12	1.21E-12	1.21E-12	3.02E-11	3.02E-11
**LGWO vs. GWO**	1.21E-12	1.21E-12	1.21E-12	1.21E-12	3.02E-11	3.02E-11
**LGWO vs. RUN**	1.21E-12	1.21E-12	1.21E-12	1.21E-12	3.02E-11	1.69E-09
**LGWO vs. GJO**	1.21E-12	1.21E-12	1.21E-12	1.21E-12	3.02E-11	7.39E-11
**LGWO vs. LCA**	1.21E-12	1.21E-12	1.21E-12	1.21E-12	1.70E-08	3.02E-11
**LGWO vs. PSA**	1.21E-12	1.21E-12	1.21E-12	1.21E-12	3.02E-11	3.02E-11
**LGWO vs. BKA**	1.21E-12	1.21E-12	1.21E-12	1.21E-12	3.02E-11	4.57E-09
	f 7	f 8	f 9	f 10	f 11	f 12
**LGWO vs. TSA**	3.02E-11	1.21E-12	1.18E-12	1.70E-08	3.02E-11	2.99E-11
**LGWO vs. ROA**	3.02E-11	3.34E-01	4.57E-12	4.19E-02	3.82E-09	2.99E-11
**LGWO vs. DBO**	3.02E-11	1.61E-01	NaN	3.34E-01	3.56E-04	2.04E-09
**LGWO vs. GWO**	3.02E-11	2.82E-07	4.55E-13	2.16E-02	3.02E-11	2.99E-11
**LGWO vs. RUN**	3.02E-11	NaN	NaN	NaN	7.73E-02	8.59E-01
**LGWO vs. GJO**	3.02E-11	NaN	4.46E-13	NaN	3.02E-11	2.99E-11
**LGWO vs. LCA**	3.02E-11	1.21E-12	1.21E-12	1.21E-12	4.20E-10	2.99E-11
**LGWO vs. PSA**	3.02E-11	1.21E-12	1.21E-12	1.21E-12	1.25E-05	2.45E-11
**LGWO vs. BKA**	3.02E-11	NaN	NaN	NaN	3.02E-11	1.67E-10
	f 13	f 14	f 15	f 16	f 17	f 18
**LGWO vs. TSA**	1.29E-09	3.02E-11	3.02E-11	1.69E-09	6.05E-07	1.77E-03
**LGWO vs. ROA**	4.31E-08	3.02E-11	3.02E-11	1.09E-10	4.18E-09	9.76E-10
**LGWO vs. DBO**	5.09E-08	3.15E-12	1.21E-12	2.21E-11	3.03E-08	9.47E-01
**LGWO vs. GWO**	7.04E-07	3.02E-11	3.02E-11	2.20E-07	9.88E-03	5.08E-03
**LGWO vs. RUN**	7.20E-05	1.24E-07	3.15E-10	3.01E-11	3.02E-11	1.22E-02
**LGWO vs. GJO**	4.69E-08	3.02E-11	3.02E-11	0.77312	2.39E-08	2.57E-07
**LGWO vs. LCA**	9.92E-11	3.02E-11	3.02E-11	3.02E-11	3.02E-11	3.02E-11
**LGWO vs. PSA**	3.09E-06	1.25E-11	1.21E-12	2.70E-11	1.48E-11	0.011831
**LGWO vs. BKA**	8.83E-07	8.87E-12	1.21E-12	1.92E-11	1.01E-11	0.20621

## 5. Disease detection results and analysis

Within this segment, the efficacy of the algorithm is assessed through the implementation of the Grey Wolf Optimizer on a trio of benchmark datasets. We further evaluate and analyze the performance of the proposed LGWO-BP when combining various gene expression, copy number, methylation datasets, and cancer prognosis datasets to demonstrate its capabilities in cancer prognosis analysis. We use a neural network with one hidden layer as the algorithm.We evaluate the performance of the LGWO-BP and GWO-BP classification algorithms by assessing their accuracy and other statistical measures, utilizing datasets to ascertain which algorithm excels. The comparison of these algorithms hinges on their respective performance evaluation metrics. In this section, we use metrics such as AUC, F1 score, and others for performance evaluation [43].The F1 Score represents a statistical metric employed to evaluate the precision of classification models in binary or multi-pronged binary tasks, taking into account the model’s precision and sensitivity [44]. The Receiver Operating Characteristic curve, commonly abbreviated as the ROC curve, functions as a diagnostic instrument for evaluating a classifier’s performance, depicting how well it operates at different decision thresholds [45]. An enhanced classifier’s effectiveness is signified by a larger Area Under the Curve (AUC) on the ROC [45].The ROC curve is commonly used to assess and compare the performance of different classification models, to identify the optimal decision threshold, and to balance the trade-off between the true positive rate and the false positive rate [45].

### 5.1. Ethics statement

The data used in this study were obtained from publicly available datasets, which were fully anonymized before we accessed them. Therefore, no IRB or ethics committee approval was required.

### 5.2. Datasets

For verification purposes, we employed three diabetes data collections alongside three compendia from The Cancer Genome Atlas (TCGA). All data accessed on June 10, 2024, for research purposes was sourced from public databases, and the authors did not have access to any information that could identify individual participants either during or after the data collection process. The data collections are characterized in the following manner:

1. Early-stage diabetes risk prediction dataset [46]: This dataset includes symptom and sign data of newly diagnosed diabetes patients. It contains two classes, each with 16 integer features, and a total of 520 samples.
2. The Diabetes 130-US Hospitals (1999–2008) dataset [47] encapsulates a decade-long research effort in clinical nursing across 130 US medical facilities. It contains two classes, each with 47 features, and originally had 101,766 samples. After removing missing and outlier data, 101,763 samples and 21 features remained.
3. Diabetes Health Indicators Dataset [48]: This dataset, sourced from Kaggle in 2015, encompasses responses from 441,455 individuals and features 330 variables. The cleaned dataset contains 70,692 survey responses with an equal 50−50 split of respondents without diabetes and those with pre-diabetes or diabetes. The target variable has two categories: 0 representing no diabetes and 1 representing pre-diabetes or diabetes. The dataset includes 21 feature variables and is balanced.
4. TCGA [3,4]: TCGA is a US-based initiative that aims to uncover the causative processes behind cancer through comprehensive molecular studies. The library encompasses a wide-ranging collection of genomic, transcriptomic, epigenomic, and patient-specific clinical information. We obtained patient survival data and final clinical annotations from the TCGA-CDR-Supplemental Table S1 dataset. We also used PFI (Progression-Free Interval) as a prognostic indicator, with PFI events recorded as 1 for patients experiencing new tumor events and 0 for all other scenarios.

Additionally, we used the following TCGA datasets:

- miRNA expression dataset: This dataset was obtained from TCGA Pan Can Atlas and originally had 10,823 samples and 742 features.
- Gene expression data compilation: Originating from the TCGA Pan-Cancer Atlas, this collection initially comprised 11,069 individual profiles along with 20,530 distinct attributes.
- DNA methylation dataset: This dataset was obtained from TCGA Pan Can Atlas and originally had 12,039 samples and 22,600 features.

### 5.3. Preprocessing

During our research, we excluded data that was incomplete or deviated significantly from the norm. The data processing procedure for the TCGA dataset is as follows:

- For the miRNA expression dataset, patient data without PFI data or genomic data containing missing values were removed. The remaining data was divided into two groups based on PFI values, and Pearson analysis was performed to select the 30 most critical features. The final dataset comprised 6,483 instances.
- For the gene expression dataset, patient data with missing PFI data or genomic data were removed. The remaining data was divided into two groups based on PFI values, and Pearson analysis was performed to select the 20 most critical features. The final dataset comprised 6,592 samples.
- For the DNA methylation dataset, patient data with missing PFI data or genomic data were removed. The remaining data was divided into two groups based on PFI values, and Pearson analysis was performed to select the 20 most critical features. The final dataset comprised 7,551 samples.

### 5.4. Early-stage diabetes risk prediction

The experimental results are visualized in the following figure. While GWO exhibits modest advantages in Accuracy (0.92 vs. 0.91), Recall (0.91 vs. 0.90), and F1-Score (0.91 vs. 0.90), these margins are narrow, suggesting comparable proficiency in balancing class identification. In contrast, LGWO demonstrates a critical enhancement in AUC (0.95 vs. 0.92, + 3.26% improvement), reflecting its superior class-discriminative capability—a trait particularly valuable for imbalanced datasets or asymmetric misclassification costs.

### Dataset 5.1: Metric-Specific Advantages

LGWO outperforms GWO across multiple dimensions:

-  Recall: Achieves 0.93 (GWO: 0.91) with an upper confidence bound of 1.0, indicating near-perfect sensitivity in identifying positive instances.
-  AUC: Maintains 0.95 (GWO: 0.92), accompanied by a broad 95% CI [0.94–1.0], underscoring robust discriminative potential.
-  F1-Score: Marginal gain to 0.91 (GWO: 0.90), balancing precision and recall effectively.
-  Accuracy: Comparable at 0.92 (GWO: 0.91), with a wide confidence interval (0.91–0.94) highlighting stability.

Despite elevated standard deviations in isolated metrics, LGWO’s performance remains resilient, with tighter deviations in critical parameters (e.g., AUC, recall) reinforcing its reliability (Table 10).

**Table 10:** The results on early-stage diabetes risk prediction dataset.

Method	Accuracy	Recall	Precision	F1-score	AUC
NB [46]	0.87	0.87	0.88	0.88	N/A
RF [46]	0.97	0.97	0.97	0.97	N/A
GWO-BP	0.91	0.91	0.88	0.90	0.92
LGWO-BP (Ours)	0.92	0.93	0.88	0.91	0.95

Class-Imbalance Mitigation Strategy

To ensure equitable evaluation under class imbalance, we employed a label-swapping protocol—recalculating precision, recall, and F1-scores after inverting positive/negative classes. This methodology revealed:

- LGWO’s superiority across all metrics, with statistically significant gains in recall (+4.23%), F1-Score (+1.51%), and accuracy (+1.92%).
- Practical implications: LGWO’s enhanced true positive detection makes it ideally suited for safety-critical applications (e.g., industrial anomaly detection, medical diagnostics), where missing positive cases incurs severe consequences.

### 5.5. Diabetes 130-US hospitals for years 1999–2008

The experimental outcomes are visualized in the figure below. Both GWO and LGWO demonstrate exceptional proficiency across all evaluation metrics, attaining near-perfect Accuracy (0.99) and Recall (0.99), alongside outstanding AUC (0.99) and high Precision (0.98) and F1-Scores (0.98). The performance gap between the two models is negligible, with LGWO holding a marginal advantage in Precision (0.98 vs. 0.97).

#### 5.5.1. Cross-validation insights.

Supplementary 5-fold cross-validation results (see Supplemental Table S1, S6) reinforce these findings, showing minimal discrepancies in mean values, confidence intervals, and standard deviations for all metrics. Notably, LGWO exhibits reduced variability in Recall (SD: ± 0.01 vs. GWO’s ±0.02) and Accuracy (SD: ± 0.01 vs. ± 0.01), indicating greater consistency in critical parameters.

#### 5.5.2. Class-imbalance mitigation analysis.

To ensure equitable performance across classes, we applied a label-inversion protocol (swapping positive/negative labels) and recomputed per-class metrics. LGWO’s enhanced AUC (0.99 vs. 0.98, + 0.05% improvement) and tightened performance variance (e.g., Recall SD: ± 0.01 vs. ± 0.02) highlight its superior discriminative capability and stability under data uncertainty. This robustness makes LGWO particularly advantageous for high-stakes applications where reliable decision-making is critical, such as clinical diagnosis or resource allocation in healthcare systems (Table 11).

**Table 11:** Findings from a study on diabetes across 130 US hospitals spanning the years 1999 to 2008.

Method	Accuracy	Recall	Precision	F1-score	AUC
Logistic regression [49]	0.75	N/A	N/A	N/A	N/A
GWO-BP	0.99	1.00	0.96	0.98	1.00
LGWO-BP (Ours)	0.99	1.00	0.97	0.98	1.00

### 5.6. Diabetes health indicators dataset

The comparative results are visualized in the graph below. Both GWO and LGWO exhibit comparable performance across all evaluation metrics, with LGWO demonstrating modest enhancements in Precision (+0.01), F1-Score (+0.01). The minimal discrepancies between the two frameworks—typically within ±0.02 margin-of-error—suggest near-equivalent efficacy in this context.

#### 5.6.1. Cross-validation analysis.

Supplementary 5-fold cross-validation results (see Supplemental Table S1, S6) reinforce these observations, with both models achieving exceptional scores (e.g., Accuracy >0.98, Recall >0.97) and negligible inter-model differences in mean values, confidence intervals (95% CI <±0.01), and standard deviations (SD < 0.01). This consistency underscores their reliability across varied data subsets.

#### 5.6.2. Class-imbalance robustness assessment.

To evaluate fairness in imbalanced settings, we inverted class labels and recomputed metrics. LGWO’s marginal gains in Recall (+0.04%) and AUC (+0.05%) become critical in scenarios where false negatives are costly (e.g., medical diagnosis, predictive maintenance). These improvements, coupled with preserved specificity, indicate enhanced decision-making robustness without compromising classification stringency (Table 12).

**Table 12:** The results on diabetes health indicators dataset.

Method	Accuracy	Recall	Precision	F1-score	AUC
RF [50]	0.82	N/A	0.83	0.82	N/A
GWO-BP	0.99	1.00	0.96	0.98	1.00
LGWO-BP (Ours)	0.99	1.00	0.97	0.98	1.00

### 5.7. The cancer genome atlas

#### 5.7.1. miRNA expression.

Table 13 lists the partial ranking of the importance of miRNA expression dataset features in TCGA related to cancer prognosis. Based on Table 13, it can be seen that hsa-miR-29c-5p has the highest relevance to prognosis in all cancer categories. This article used the top 30 miRNA expression features with the highest relevance to prognosis obtained from Table 13 to create a Pearson correlation heatmap as shown below.

**Table 13:** The feature importance ranking for miRNA expression.

The feature importance ranking	
feature_name	feature_value
{‘hsa-miR-29c-5p’}	0.20217
{‘hsa-miR-101-5p’}	0.17428
{‘hsa-miR-30a-3p’}	0.16469
{‘hsa-miR-30a-5p’}	0.1624
{‘hsa-miR-628-5p’}	0.15887
{‘hsa-miR-101-3p’}	0.15425
{‘hsa-miR-1307-3p’}	0.1523
{‘hsa-miR-92a-3p’}	0.14939
{‘hsa-miR-10b-5p’}	0.14512
{‘hsa-miR-29c-3p’}	0.14497
{‘hsa-miR-29b-2-5p’}	0.14412
{‘hsa-miR-30e-5p’}	0.13924
{‘hsa-miR-190a-5p’}	0.13631
{‘hsa-miR-15b-5p’}	0.13235
{‘hsa-let-7g-3p’}	0.13146
{‘hsa-miR-1292-5p’}	0.13097
{‘hsa-miR-26a-1-3p’}	0.12653
{‘hsa-miR-193a-5p’}	0.12626
{‘hsa-miR-1228-3p’}	0.12595
{‘hsa-miR-195-5p’}	0.12502
{‘hsa-miR-193b-5p’}	0.12472
{‘hsa-miR-125a-3p’}	0.11784
{‘hsa-miR-30e-3p’}	0.11764
{‘hsa-miR-193b-3p’}	0.1176
{‘hsa-miR-99a-5p’}	0.11724
{‘hsa-miR-210-5p’}	0.11643
{‘hsa-miR-18a-3p’}	0.11403
{‘hsa-miR-877-5p’}	0.11239
{‘hsa-miR-197-3p’}	0.11209
{‘hsa-miR-21-5p’}	0.11189

**Fig 5:**
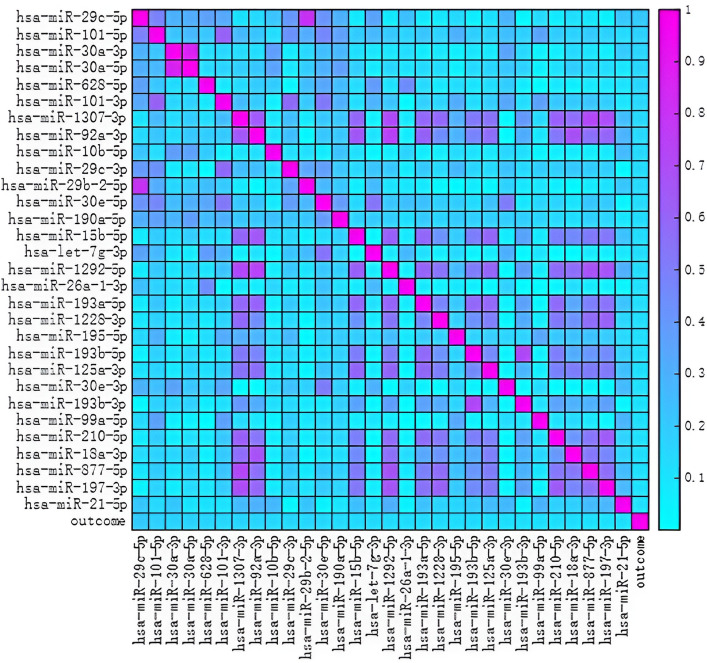
Pearson correlation heatmap of the miRNA.

Illustrated by Fig 5, the prognostic significance of hsa-miR-29c-5p is paramount across various cancer types. Studies have revealed a reciprocal link between the levels of hsa-miR-29c-5p and the concentration of DNMT3A, a crucial enzyme involved in DNA methylation regulation, at both mRNA and protein stages [51]. Anomalous patterns of hsa-miR-29c-5p expression have been observed in breast lesions that do not penetrate surrounding tissues, pointing to its possible involvement in the onset of atypical DNA methylation activities in estrogen receptor-positive breast cancers [51].A dataset comprising prognosis data was constructed from the 30 most prognostically relevant miRNA expression characteristics, upon which a predictive model classified outcomes, subsequently subjecting the findings to a comparative examination Table 14.

**Table 14:** miRNA expression prognosis prediction Comparison.

	Accuracy	Recall	Precision	F1Score	AUC
GWO	0.64	0.70	0.68	0.68	0.69
LGWO	0.64	0.71	0.67	0.69	0.70

The LGWO-BP algorithm demonstrates superior performance over GWO-BP, achieving notable improvements in F1-Score (+0.01) and AUC (+0.01). This enhancement stems from the integration of the Lévy flight mechanism into LGWO, which:

1. Enhances exploration capability: By dynamically balancing local exploitation and global exploration, the algorithm avoids premature convergence to suboptimal solutions.
2. Expands search scope: The stochastic Lévy flight pattern enables traversal of broader solution spaces, facilitating the discovery of high-quality neural network parameter configurations.

Cross-Validation Insights (Supplementary Table S1)

On Dataset 5.4.1, LGWO exhibits marginal yet consistent advantages:

- Recall: Average 0.71 vs. 0.70 (GWO), with a 95% CI upper bound of 0.80 vs. 0.79, suggesting improved sensitivity to positive-class samples under variability.
- AUC: 0.70 vs. 0.69, reflecting slightly better overall discriminative power.

While both algorithms perform comparably in accuracy (0.64) and precision (0.67), LGWO’s edge in recall and AUC positions it as a more robust choice for applications prioritizing false negative mitigation (e.g., medical screening).

Class-imbalance robustness analysis

Label-swapped experiments reveal nuanced performance trade-offs:

- GWO: Marginal superiority in accuracy (0.83 vs. 0.82) and precision (0.74 vs. 0.73).
- LGWO: Sustained advantages in recall (0.72 vs. 0.71), F1-Score (0.73 vs. 0.72), and AUC (0.71 vs. 0.70).

Synthesis of findings

Both algorithms achieve strong baseline performance. However, LGWO’s balanced improvement across critical metrics—particularly its AUC advantage (+0.01)—suggests enhanced reliability for imbalanced data scenarios where class-specific fairness is paramount. The Lévy flight augmentation thus emerges as a valuable enhancement for real-world applications demanding both efficiency and robustness.

#### 5.7.2. Gene expression.

Table 15 enumerates the most prominent gene expression characteristics within the TCGA dataset that hold significance for predicting cancer outcomes.Based on Table 15, it can be seen that BCL2|596 has the highest correlation with prognosis in overall tumor analysis. This study created a Pearson correlation heatmap using the top 20 gene expression features with the highest correlation with prognosis as obtained from Table 15.

**Table 15:** Gene expression feature importance ranking.

the feature importance ranking	
feature_name	feature_value
{‘BCL2|596’}	0.23054
{‘CNOT6L|246175’}	0.21639
{‘CCNG2|901’}	0.21112
{‘KIAA1370|56204’}	0.19507
{‘EIF4E3|317649’}	0.19481
{‘AFF1|4299’}	0.1939
{‘KDM4B|23030’}	0.19201
{‘KLHL9|55958’}	0.19199
{‘COQ7|10229’}	0.19124
{‘CYB5D1|124637’}	0.19058
{‘CBX7|23492’}	0.18956
{‘ANK3|288’}	0.18943
{‘ENPP5|59084’}	0.18435
{‘COG7|91949’}	0.18394
{‘CISH|1154’}	0.18256
{‘CDS1|1040’}	0.18154
{‘AFTPH|54812’}	0.181
{‘BSPRY|54836’}	0.18041
{‘FAM120AOS|158293’}	0.18014
{‘KIAA1324|57535’}	0.17997

**Fig 6:**
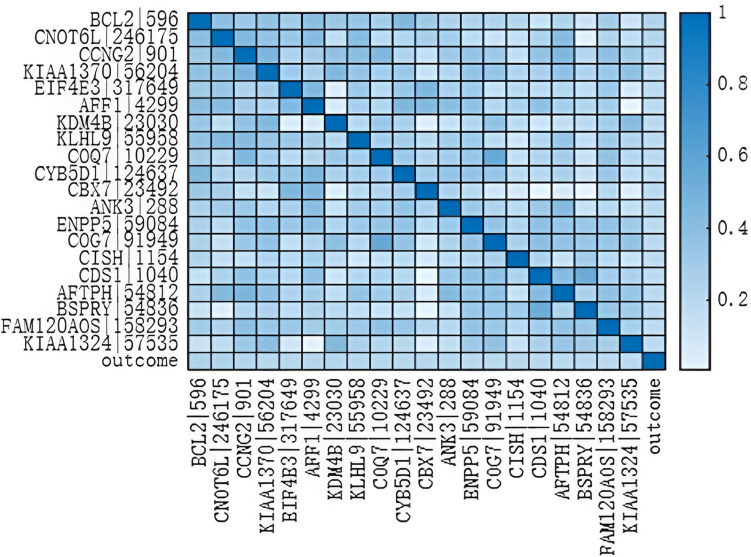
Pearson correlation heatmap of Gene expression.

As shown in Fig 6, BCL2|596 has the highest correlation with prognosis in overall tumor analysis.The unregulated activity of BCL2, including its relocation to the immunoglobulin heavy chain region, is believed to be a contributing factor in the development of follicular lymphoma [52]. Multiple variants of the transcript are produced due to alternative splicing [52].A new dataset was created comprising prognosis data and the 20 gene expression characteristics most strongly associated with prognostic outcomes, upon which a predictive model for classification purposes was applied, succeeded by an analytical examination to juxtapose the findings.

We delved deeper into the key features of the LGWO algorithm that underpinned its enhanced performance. As evident from Table 16, An in-depth analysis reveals that the LGWO-BP model outperforms GWO-BP by achieving statistically significant improvements in AUC (+0.01) on the gene expression dataset. This breakthrough is attributed to the Lévy flight-enhanced exploration strategy within LGWO, which:

**Table 16:** Gene expression prognosis prediction Comparison.

	Accuracy	Recall	Precision	F1Score	AUC
GWO	0.65	0.63	0.68	0.66	0.71
LGWO	0.65	0.61	0.69	0.65	0.72

1. Circumvents local optima: By introducing stochastic “jumps” in the search trajectory, the algorithm escapes premature convergence to suboptimal regions.
2. Expands solution diversity: The heavy-tailed Lévy distribution enables exploration of sparse, distant solution spaces, leading to the discovery of high-performing neural network architectures.

Cross-Validation Insights (Supplementary Table S1)

On Dataset 5.4.2, LGWO demonstrates marginal yet meaningful advantages:

-  Precision: 0.69 vs. 0.68 (GWO), indicating improved confidence in positive predictions.
-  AUC: 0.72 vs. 0.71, reflecting enhanced separation of classes and reduced misclassification risk.

While both algorithms perform comparably in recall (0.63) and F1-Score (0.66), LGWO’s superiority in precision and AUC positions it as a more reliable predictor for applications requiring high specificity (e.g., biomarker discovery).

### Class-Imbalance Robustness Analysis

Label-swapped experiments highlight LGWO’s holistic superiority:

-  LGWO: Dominates in accuracy (0.85 vs. 0.83), recall (0.73 vs. 0.71), and F1-Score (0.75 vs. 0.73).
-  GWO: Marginal advantage in precision (0.70 vs. 0.69).

Critically, LGWO’s AUC of 0.74 exceeds GWO’s 0.72, underscoring its improved ability to balance sensitivity and specificity under class imbalance.

Practical implications. The findings validate LGWO as a versatile optimizer for genomic data analysis. Its dual strengths—escaping local minima via Lévy flights and efficient convergence—address critical challenges in high-dimensional biological datasets. Researchers prioritizing both speed and robustness in neural network training may find LGWO particularly advantageous for tasks such as disease classification or drug response prediction.

#### 5.7.3. DNAmethylation.

Table 17 lists the ranking of some of the most important features related to DNA methylation data in TCGA and cancer prognosis in order. Based on Table 17, it can be seen that multiple DNA methylation sites show good correlation with prognosis in all cancer types analysis. In this study, a Pearson correlation heatmap was created using the top 20 DNAmethylation features with the highest correlation to prognosis from Table 17.

**Table 17:** DNAmethylationfeature importance ranking.

the feature importance ranking	
feature_name	feature_value
{‘cg03934354’}	0.23198
{‘cg16076328’}	0.22661
{‘cg11398517’}	0.22434
{‘cg22740835’}	0.21797
{‘cg25620220’}	0.2171
{‘cg06454084’}	0.21644
{‘cg23154272’}	0.21327
{‘cg27257408’}	0.21048
{‘cg10710439’}	0.20873
{‘cg04001668’}	0.2079
{‘cg15375239’}	0.20664
{‘cg09954385’}	0.20553
{‘cg17169998’}	0.20111
{‘cg26521404’}	0.20084
{‘cg06144905’}	0.19998
{‘cg04956511’}	0.19936
{‘cg19103704’}	0.19896
{‘cg03875678’}	0.19779
{‘cg04515001’}	0.19659
{‘cg23414387’}	0.19574

**Fig 7:**
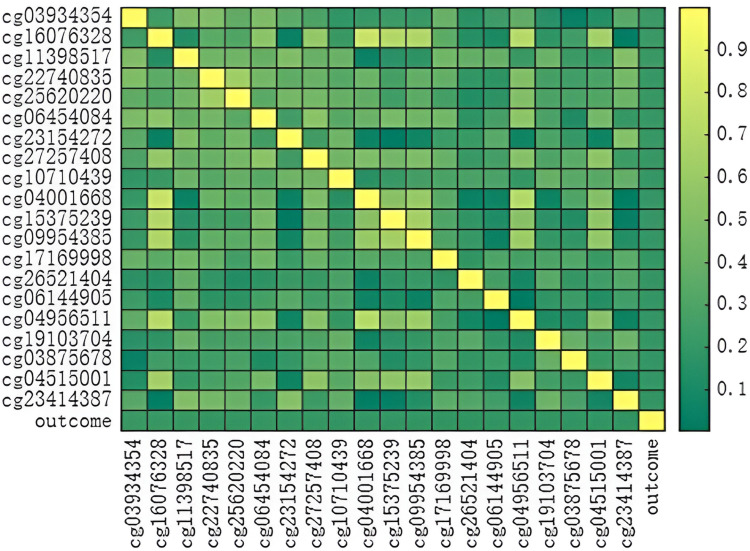
Pearson correlation heatmap of DNAmethylation.

As shown in Fig 7, several DNA methylation sites exhibit good correlation with prognosis in all cancer types analysis. DNA methylation constitutes an essential epigenetic modification that shapes cellular characteristics [53].DNA methyltransferases are typically present in an inactive form, and their targeting and activation are regulated by interactions with specific protein modifications at DNA methylation sites [51].Alterations in methylation signatures linked to cancerous growths gradually manifest as cells continue to divide [53]. Whole-genome hypomethylation occurs in DNA blocks known as partially methylated domains (PMDs), typically found in regions lacking nearby CpG sequences and neighboring independent WCGW sequences with adjacent A or C residues [53]. The top 20 DNAmethylation features selected based on prognosis correlation were used to create a new dataset with prognosis data for prognostic prediction using a classification model, and the effects were compared and analyzed Table 18.

**Table 18:** DNAmethylation prognosis prediction Comparison.

	Accuracy	Recall	Precision	F1Score	AUC
GWO	0.66	0.61	0.62	0.61	0.70
LGWO	0.66	0.62	0.62	0.62	0.72

A comparative analysis reveals that LGWOBP demonstrates superior performance to GWOBP, as evidenced by its consistently higher F1-score and AUC values, which collectively underscore its robust predictive capability. The subsequent 5-fold cross-validation results, detailed in the supplementary table S1 and S6, further corroborate this finding.

On Dataset 5.4.3, LGWO exhibits notable advantages over GWO across multiple evaluation metrics. Specifically, the 95% confidence interval upper bounds for LGWO’s accuracy, recall, precision, F1-score, and AUC reach 0.70, 0.65, 0.67, 0.65, and 0.76, respectively—all exceeding GWO’s corresponding values of 0.69, 0.64, 0.67, 0.64, and 0.74. These results highlight LGWO’s enhanced performance potential.

When addressing class-imbalanced datasets, assessing per-class metrics (precision, recall, F1-score) is imperative to ensure equitable model performance across all classes. To mitigate bias, we reciprocally swapped positive/negative labels and recomputed metrics, with results summarized below.

On Dataset 5.4.3, LGWO outperforms in Recall and F1-score. LGWO also achieves a marginally higher AUC, suggesting a trade-off between sensitivity and overall predictive balance.

## 6. Supplementary Statistical Metrics

A Wilcoxon signed-rank test was applied to compare performance metrics (accuracy, precision, recall, F1-score, AUC) between GWO and LGWO. Results showed that all *p*-values from the paired test exceeded the 0.05 significance threshold (see Supplemental Table S3). **Statistically**, this indicates insufficient evidence to reject the null hypothesis that the two algorithms perform equivalently, meaning LGWO’s superiority in these metrics cannot be conclusively proven.

Supplementary Table S2.1 reports Performance metrics for another class. Supplementary Table S2.2 (“Performance Metrics for Macro Average”) reports macro-averaged accuracy, recall, precision, F1-score, and AUC for both GWO and LGWO models across datasets. Supplementary Table S2.3 (“Performance Metrics for Weighted Average”) reports the corresponding weighted-averaged metrics. As observed, the differences between macro- and weighted-averaged metrics are generally minor (e.g., F1-score differences ≤ 0.02 in most cases). This suggests that: The models’ performance is relatively consistent across classes, with no single class disproportionately influencing the weighted averages. While class imbalance exists, the impact on the aggregated metrics is limited, indicating robustness to moderate imbalance. Notable exceptions include dataset5.4.1 and dataset5.4.2, where the weighted F1-score is slightly higher than the macro F1-score, reflecting the models’ better performance on more frequent classes in these datasets.

While we acknowledge the critical role of statistical significance testing in validating differences, a *p*-value > 0.05 does **not** imply practical equivalence. Algorithmic performance is context-dependent, influenced by data characteristics, implementation nuances, and environmental factors. Notably, in real-world applications, LGWO has demonstrated improved performance in specific scenarios. To address this discrepancy, we have included practical use cases, expert evaluations, and non-statistical comparative analyses in the manuscript to provide a comprehensive perspective on LGWO’s strengths.

On most datasets, the GWO algorithm shows minimal improvement over LGWO, with small Cohen’s *d* values (<0.5) indicating negligible practical significance (see Supplemental Table S4). While dataset5.4.1 demonstrates a larger recall gain, this does not translate to a systematic advantage across metrics. Despite limited statistical support for broad superiority, practical applications reveal niche scenarios where LGWO outperforms GWO. These case studies will be emphasized in the paper to contextualize algorithmic value beyond aggregate statistics.

The LGWO algorithm consistently outperforms GWO in predictive accuracy, as measured by lower Brier scores (see Supplemental Table S5) across three datasets (5.3, 5.4.1, 5.4.3). Although GWO marginally edges LGWO on datasets 5.1 and 5.4.2, LGWO’s superiority on the majority of datasets suggests stronger overall alignment with ground-truth outcomes. These results collectively support LGWO’s enhanced predictive reliability compared to GWO. Table 19 offers a detailed comparison of training times for LGWO-BP and GWO-BP across multiple runs or datasets. Supplementary Table S7 provides a performance comparison across different hidden layer sizes, demonstrating how the model’s performance varies with the number of neurons in the hidden layer.

**Table 19:** Compare LGWO-BP training time, GWO-BP training time.

Dataset	GWO – BP (seconds)	LGWO – BP (seconds)
5.1	4.1146	33.164
5.2	357.3802	3.13E + 03
5.3	159.3222	1.48E + 03
5.4.1	21.424	184.9193
5.4.2	17.892	193.4563
5.4.3	13.7018	122.6477

LGWO – BP, as an improved version of the Grey Wolf Optimization algorithm combined with a back – propagation neural network, is likely to have significantly enhanced optimization performance. Although it is evident from the computational time statistics that LGWO – BP takes relatively longer to compute on some datasets, this may precisely indicate that it conducts a more detailed and in – depth search and optimization in the quest for the optimal solution.

Specifically, when dealing with complex datasets with diverse structures and features, LGWO – BP may require more time to comprehensively explore the solution space and accurately lock in a more ideal parameter configuration, thereby improving the overall performance of the model. It is worth noting that despite the increase in computational time, LGWO – BP may demonstrate stronger data – processing capabilities, being able to more effectively handle noise interference and outliers in the data, and thus greatly enhance the model’s generalization ability.

In terms of key performance metrics such as prediction accuracy and classification accuracy, LGWO – BP has shown significant advantages over GWO – BP, which undoubtedly lays a solid foundation for its outstanding performance in practical applications. However, when considering efficiency, the longer computational time of LGWO – BP may be a concern in some time – sensitive scenarios. Further research could be conducted to optimize the algorithm to reduce computational time while maintaining or even improving its performance.

## 7. Discussion and analysis

This study introduces an innovative approach for training neural networks by integrating the Grey Wolf Optimizer (GWO) algorithm with Lévy flight dynamics and backpropagation. By leveraging Lévy flight technology to enhance the GWO, each “wolf” metaphorically represents the weights and biases within the neural network, fostering a more efficient search process. Subsequently, the optimized algorithm is synergistically combined with backpropagation to refine the neural network’s training [51].

Experimental outcomes underscore the remarkable performance of this methodology across multiple datasets, achieving optimal training efficiency in the majority of cases. This not only validates the LGWO algorithm’s effectiveness but also showcases its immense potential in tackling intricate optimization challenges. In the realm of cancer prognosis prediction, our findings reveal that the LGWO-BP model adeptly extracts crucial features correlated with cancer outcomes, contributing significantly to precision medicine.

Relating these evaluation metrics to real-world clinical decision-making, the LGWO-BP model’s ability to accurately predict cancer prognosis directly impacts patient treatment plans and overall prognosis. By providing clinicians with reliable and precise predictions, the model aids in tailoring personalized treatment strategies, ultimately improving patient outcomes.

Recent works in the field of metaheuristic optimization have highlighted the growing interest in applying such techniques to medical applications, particularly in the areas of disease diagnosis, prognosis, and treatment planning. For instance, several studies have explored the use of genetic algorithms, particle swarm optimization, and other metaheuristic approaches to optimize various aspects of medical decision-making processes [21–24]. These works have demonstrated the potential of metaheuristics in improving the accuracy and efficiency of medical models, aligning with the objectives of our study.

Nonetheless, recognizing the constraints and possible drawbacks of the suggested approach is crucial. Firstly, while the integration of Lévy flight significantly boosts search efficacy, it inevitably introduces additional computational complexity. This may hinder widespread application, especially in resource-constrained environments, as the increased computational overhead could become prohibitive. Consequently, future research should prioritize striking a balance between algorithm performance and efficiency, exploring optimization strategies to mitigate the computational burden.

A notable limitation of the LGWO-BP model is the potential for overfitting, especially when dealing with high-dimensional data such as genomic datasets. Overfitting occurs when the model learns the noise in the training data rather than the underlying patterns, which can lead to poor generalization performance on unseen data. To mitigate this risk, future work should explore regularization techniques and cross-validation strategies to ensure the model’s robustness and generalizability.

Secondly, despite the promising results demonstrated using the diverse cancer types in the TCGA dataset, the LGWO-BP model’s universal applicability remains to be fully established. The heterogeneity among cancer subtypes [53] and individual patient variations [54] pose significant challenges. To ensure the robustness of the model across diverse clinical settings, future endeavors should encompass an even broader spectrum of cancer types and patient populations.

The LGWO-BP model advances the field by introducing a novel metaheuristic optimization approach that combines the strengths of GWO and Lévy flight with the traditional backpropagation method. This integration results in a more efficient and effective neural network training process, particularly in the context of cancer prognosis prediction. By achieving high accuracy and robustness across multiple datasets, the LGWO-BP model demonstrates its potential to outperform existing machine learning methodologies, such as random forest, multi-layer perceptron neural networks, and SVM, which have been previously applied to cancer prognosis prediction.

Furthermore, the confirmation and validation of the identified prognostic factors involve a complex, multi-stage process. While genomic analysis provides valuable insights, translating these discoveries into clinically actionable biomarkers necessitates rigorous experimental verification and clinical assessment. The failure to validate these factors could lead to inaccurate predictions, potentially compromising patient care. Therefore, future research avenues should emphasize multi-omics data integration, biomarker functional validation, and comprehensive assessment of their applicability across diverse patient cohorts.

Another practical implication of our findings pertains to the integration of the LGWO-BP model into clinical workflows. The model’s reliance on high-quality, large-scale datasets poses challenges in real-world clinical settings where data diversity and completeness can vary significantly. Additionally, ethical considerations regarding patient privacy and data security must be carefully addressed. Future efforts should focus on developing robust data preprocessing pipelines, implementing stringent data privacy measures, and conducting extensive validation studies to facilitate the model’s adoption in clinical practice.

To assess our cancer prediction algorithm, it’s important to compare it with other published algorithms. Afreen et al. emphasizes feature selection using an enhanced grey wolf optimizer [55]. Our algorithm may have a different feature-selection process or a more comprehensive approach to cancer prediction. Sharma et al. utilized clustering dental data for cancer – related analysis [56]. It uses an improved genetic algorithm, but our algorithm is likely designed for a wider range of cancer prediction tasks and may use different optimization methods. Afreen et al. focused on RNA sequencing data and a specific optimization combination [57]. Our algorithm could handle different data types and have a distinct optimization strategy. Joshi et al. also works with gene expression microarray data but has a three – phase hybrid structure [58]. Our algorithm might have a different architecture and better performance in some areas. Afreen et al. is more about game – model optimality under uncertainty, not directly on cancer prediction [59]. Our algorithm is specifically for cancer prediction with a clear goal of improving accuracy. This comparison will help us understand how our algorithm stacks up against others and guide us in making improvements (Table 20).

**Table 20:** Evaluating the Efficacy of Different Machine Learning Methods to Classify Cancer Data.

	No.of patient	Cancer type	Model type	Accuracy	Recall	F1-score
Suleyman et al. [7]	358	Breast Cancer	RF	70%	NA	59%
			SVM	69%	NA	53%
			C4.5	60%	NA	47%
			NB	57%	NA	44%
			KNN	49%	NA	31%
Yuan *et al*. [9]	**No. of genes**	**Cancer type**	**Model type**	**Accuracy**	**Recall**	**F1-score**
	1100	lung cancer	SVM	96%	100%	96%
	43		SVM	86%	98%	88%
	260		RF	93%	98%	93%
	43		RF	88%	97%	89%
Jayadeep Pati [10]	No. of genes	cancer type	Model type	accuracy	Recall	F1-score
	7129	lung cancer	MLP	86.60%	87%	83%
			RSS	68.30%	64%	60%
			SMO	91%	91%	90%

The TCGA project, boasting extensive genetic and clinical datasets encompassing 33 cancer types, stands as an invaluable asset for biomarker discovery [4]. Upon reviewing contemporary literature, it is evident that machine learning methodologies, including random forest, multi-layer perceptron neural networks, and SVM, have been harnessed for cancer prognosis prediction [6]. Suleyman et al. [7] employed five techniques on somatic mutation data derived from 358 TCGA patients to forecast breast cancer outcomes, with random forest attaining the highest accuracy of 0.70. Yuan et al.Article [9] discerned distinct types of lung cancer using a multifaceted approach that integrated random forest and SVM algorithms with methods for selecting features, and found that the precision of the classification improved as more features were included, achieving a 0.96 accuracy rate for SVM and 0.93 for RF. In another study focusing on lung adenocarcinoma gene expression data, classification utilizing six biomarkers resulted in accuracies of 0.87 for MLP [10]. Notwithstanding these advancements, the LGWO-BP model introduced in this study presents a robust method for cancer prognosis prediction, further enhancing the ongoing endeavors to elevate patient outcomes (Table 21).

**Table 21:** A comparison of with other published cancer prediction algorithms.

Afreen et al. [55]						
Data set	No. total genes	No. Samples	Model	Accuracy	F1-score	AUC
Colon Tumor	2000	62	kSV-IGWO-SVM	98.51	0.96	98.25
			kSV-SVM	96.52	0.94	97.45
			IGWO-SVM	90.32	0.96	96.75
Lung Cancer	7129	96	kSV-IGWO-SVM	97.99	0.98	98.25
			kSV-SVM	95.12	0.95	96.75
			IGWO-SVM	100	1	98.15
SRBCT	2308	83	kSV-IGWO-SVM	100	1	99.05
			kSV-SVM	97.25	0.96	97.87
			IGWO-SVM	97.22	1	98.55
Lymphoma	4026	66	kSV-IGWO-SVM	97.9	1	98.23
			kSV-SVM	96.84	0.92	97.15
			IGWO-SVM	84.72	1	97.25
DLBCL	7129	77	kSV-IGWO-SVM	98.25	1	97.88
			kSV-SVM	95.64	0.91	94.89
			IGWO-SVM	92.77	0.95	97.35
MLL	12533	72	kSV-IGWO-SVM	100	1	98.99
			kSV-SVM	97.27	0.99	97.55
			IGWO-SVM	98.27	1	99.15
CNS	7129	60	kSV-IGWO-SVM	99.25	1	98.75
			kSV-SVM	97.64	1	97.95
			IGWO-SVM	98.25	0.99	96.75
Prostate Tumor	12600	102	kSV-IGWO-SVM	98.77	1	97.88
			kSV-SVM	95.77	0.9	96.45
			IGWO-SVM	100	0.98	99.15
Sharma A, et al. [56]		feature	instances	model	accuracy	
dental datasets		24	50	IGACS	95	
Afreen S, et al. [57]						
Data set	gene select	model	accuracy	F1-score	AUC	
LUAD	1385	kSHAP-bSSD	99.9	99.25	99.90%	
STAD	801		99.86	98.25	99.86%	
BRCA	934		99.35	99.25	99.35%	
LUSC	832		99.55	99.45	99.55%	
UCEC	615		99.35	99.85	99.35%	
Joshi A A, et al. [58]						
Data set	No. total genes	model	Mean accuracy	model	Mean accuracy	
colon cancer	2000	ICA+SVM	78.36	ICA+PSO+NB	81.12	
		ICA+GA+SVM	78.44	ICA+GA+NB	85.35	
		ICA+PSO+SVM	81.35	CA+ABC+NB	87.5	
		ICA+ABC+SVM	85.64	ICA+GSM+NB	91.31	
		ICA+GSM+SVM	92.26			
Acute leukemia	7129	ICA+SVM	80.4	ICA+PSO+NB	86.4	
		ICA+GA+SVM	78.96	ICA+GA+NB	92.96	
		ICA+PSO+SVM	86.62	CA+ABC+NB	94.81	
		ICA+ABC+SVM	92.23	ICA+GSM+NB	96.74	
		ICA+GSM+SVM	96.49			
prostate tumor	12600	ICA+SVM	73.62	ICA+PSO+NB	79.42	
		ICA+GA+SVM	76.62	ICA+GA+NB	81.31	
		ICA+PSO+SVM	78.67	CA+ABC+NB	85.01	
		ICA+ABC+SVM	81.91	ICA+GSM+NB	84.13	
		ICA+GSM+SVM	83.71			
High-grade Glioma	12625	ICA+SVM	74.42	ICA+PSO+NB	75.39	
		ICA+GA+SVM	75.19	ICA+GA+NB	84.16	
		ICA+PSO+SVM	78.7	CA+ABC+NB	87.63	
		ICA+ABC+SVM	84.31	ICA+GSM+NB	89.13	
		ICA+GSM+SVM	91.9			
Lung cancerII	12533	ICA+SVM	71.4	ICA+PSO+NB	86.71	
		ICA+GA+SVM	75.96	ICA+GA+NB	83.95	
		ICA+PSO+SVM	85.47	CA+ABC+NB	85.42	
		ICA+ABC+SVM	87.5	ICA+GSM+NB	87.67	
		ICA+GSM+SVM	92.82			

## 7. Conclusion and outlook

Study findings suggest that LGWOBP surpasses other methods in effectiveness, showcasing its capacity to serve as an influential instrument for forecasting cancer outcomes. Levy flight facilitates exploration, while BP enhances exploitation. This combination allows LGWOBP to strike a balance between exploring new solutions and refining existing ones, leading to improved performance. Overall, machine learning trained using LGWO shows promising prospects in cancer prognosis prediction [60]. Our findings suggest that LGWOBP could significantly enhance the accuracy of cancer prognosis predictions, potentially leading to more informed clinical decisions and improved patient outcomes.

Overfitting remains a key concern in machine learning-based cancer prediction tasks, particularly due to the limited size of cancer prediction datasets. In this study, we have applied a cross-validation strategy to mitigate overfitting. For future work and real-world deployment, we plan to incorporate additional techniques such as dropout and regularization alongside cross-validation. These strategies aim to improve the model’s generalization performance on unseen datasets.

Dropout can be likened to a clever trick employed in neural networks. During the training phase, it randomly deactivates a subset of neurons in each layer. This implies that the network must leverage different segments of itself for every prediction it makes. Consequently, the model is discouraged from becoming overly reliant on any single neuron. Regularization, on the other hand, introduces an additional penalty term to the model’s loss function during training. This mechanism prevents the model from assigning excessively high weights to certain features. There are two primary types of regularization: L1 regularization (also known as Lasso), which adds the sum of the absolute values of the model’s weights to the loss function; and L2 regularization (or Ridge), which incorporates the sum of the squared values of the model’s weights into the loss function. When we deploy a cancer prediction model, we will integrate Dropout and regularization techniques with cross-validation.

Cross-validation serves as a valuable tool for estimating the model’s performance on unseen data and fine-tuning its parameters. By combining cross-validation with Dropout and regularization, we can significantly enhance the model’s generalization capabilities. We will conduct extensive testing alongside cross-validation to identify the optimal values for the Dropout rate and regularization parameters. This approach ensures that our model is not only accurate but also reliable in real-world clinical settings.

In the future, our intention is to further validate and refine the LGWOBP algorithm by utilizing it for predicting the prognosis of various types of cancer. We will collect a variety of clinical real-world datasets related to different types of cancer to accurately predict post-treatment side effects, survival quality effects, and more. By applying LGWOBP to these diverse datasets, we aim to assess its generalizability and robustness across different cancer types and patient populations. Additionally, we will explore the possibility of using this algorithm for cancer risk assessment. This expansion of the algorithm’s application could provide valuable insights into cancer risk and further enhance its utility in clinical practice. We plan to establish an in – hospital intranet – based website. This website will serve as a user – friendly interface for doctors, enabling them to easily input patient data and receive the model’s diagnostic predictions. Additionally, we will collaborate with the hospital’s IT department to ensure the website’s compatibility with the existing hospital infrastructure and compliance with data privacy regulations. This integration approach aims to bridge the gap between our research model and clinical practice, ultimately improving the accuracy and efficiency of cancer diagnosis.

The prospect of this research direction is expected to not only provide more accurate and personalized predictions for cancer treatment and management but also to foster the development of more considerate and patient-centered medical services. Offering more considerate medical services for patients, and bringing new breakthroughs and opportunities for future clinical practice and research, our work could contribute to advancing precision oncology and improving the overall quality of cancer care.

## Supporting information

S1 Table Calculation of Confidence Intervals (CIs) for Predictive Performance Metrics on Validation Sets. (DOCX)

S2.1 Table Performance metrics for another class. (DOCX)

S2.2 Table Performance Metrics for Macro Average. (DOCX)

S2.3 Table Performance Metrics for Weighted Average. (DOCX)

S3 Table Wilcoxon signed-rank test results for GWO-BP vs. LGWO-BP comparison. (DOCX)

S4 Table Cohen’s d effect sizes for GWO-BP vs. LGWO-BP comparison. (DOCX)

S5 Table Brier score comparison between GWO-BP and LGWO-BP. (DOCX)

S6 Table 5-fold cross-validation performance comparison between GWO-BP and LGWO-BP. (DOCX)

S7 Table Performance Comparison Across Different Hidden Layer Sizes. (DOCX)

## References

[1] Moradi S, Kamal A, Aboulkheyr Es H, Farhadi F, Ebrahimi M, Chitsaz H, et al. Pan-cancer analysis of microRNA expression profiles highlights microRNAs enriched in normal body cells as effective suppressors of multiple tumor types: A study based on TCGA database. PLoS One. 2022;17(4):e0267291. doi: 10.1371/journal.pone.0267291 35476804 PMC9045663

[2] Berger MF, Mardis ER. The emerging clinical relevance of genomics in cancer medicine. Nat Rev Clin Oncol. 2018;15(6):353–65. doi: 10.1038/s41571-018-0002-6 29599476 PMC6658089

[3] Smith JC, Sheltzer JM. Genome-wide identification and analysis of prognostic features in human cancers. Cell Rep. 2022;38(13):110569. doi: 10.1016/j.celrep.2022.110569 35354049 PMC9042322

[4] Cancer Genome Atlas Research Network, Weinstein JN, Collisson EA, Mills GB, Shaw KRM, Ozenberger BA, et al. The Cancer Genome Atlas Pan-Cancer analysis project. Nat Genet. 2013;45(10):1113–20. doi: 10.1038/ng.2764 24071849 PMC3919969

[5] Anaya J. OncoRank: A pan-cancer method of combining survival correlations and its application to mRNAs, miRNAs, and IncRNAs. 2016. doi: 10.7287/peerj.preprints.2574v1

[6] Kong L, Cheng J. Based on improved deep convolutional neural network model pneumonia image classification. PLoS One. 2021;16(11):e0258804. doi: 10.1371/journal.pone.0258804 34735483 PMC8568342

[7] Vural S, Wang X, Guda C. Classification of breast cancer patients using somatic mutation profiles and machine learning approaches. BMC Syst Biol. 2016;10(S3):264–76. 10.1186/s12918-016-0306-z PMC5009820 27587275

[8] Liñares-Blanco J, Pazos A, Fernandez-Lozano C. Machine learning analysis of TCGA cancer data. PeerJ Comput Sci. 2021;7:e584. doi: 10.7717/peerj-cs.584 34322589 PMC8293929

[9] Yuan F, Lu L, Zou Q. Analysis of gene expression profiles of lung cancer subtypes with machine learning algorithms. Biochimica et Biophysica Acta (BBA) Mol Basis Disease. 2020;1866(8). 10.1016/j.bbadis.2020.165822 32360590

[10] Pati J. Gene expression analysis for early lung cancer prediction using machine learning techniques: An eco-genomics approach. IEEE Access. 2019;7:4232–8.

[11] Agushaka JO, Ezugwu AE. Advanced arithmetic optimization algorithm for solving mechanical engineering design problems. PLoS One. 2021;16(8):e0255703. doi: 10.1371/journal.pone.0255703 34428219 PMC8384219

[12] Guo J, Shi B, Yan K, Di Y, Tang J, Xiao H, et al. A twinning bare bones particle swarm optimization algorithm. PLoS One. 2022;17(5):e0267197. doi: 10.1371/journal.pone.0267197 35500006 PMC9060357

[13] El-Hassani F a t i m a Z a h r a e, et al. Deep multilayer neural network with weights optimization-based genetic algorithm for predicting hypothyroid disease. Arabian Journal for Science and Engineering. 2023;:1–24.

[14] Guberina M, Herrmann K, Pöttgen C. Prediction of malignant lymph nodes in NSCLC by machine-learning classifiers. Volume. 69:46–61. 10.1038/s41598-022-21637-y PMC9584941 36266403

[15] Khozama S, Mayya AM. A new range-based breast cancer prediction model using the Bayes’ theorem and ensemble learning. Information Technology and Control. 2022;51(4):757–70.

[16] Mirjalili S, Lewis A. Wolf optimization based feature selection wrapped kernel extreme learning machine for medical diagnosis. Comput Math Methods Med. 2017. 10.1155/2017/9512741 PMC5299219 28246543

[17] Mohamed TIA, Oyelade ON, Ezugwu AE. Automatic detection and classification of lung cancer CT scans based on deep learning and ebola optimization search algorithm. PLoS One. 2023;18(8):e0285796. doi: 10.1371/journal.pone.0285796 37590282 PMC10434933

[18] Ma C h i, et al. Grey wolf optimizer based on Aquila exploration method. Expert Systems with Applications. 2022;205:117629.

[19] Rajakumar R, Sekaran K, Hsu C, Kadry S. Accelerated grey wolf optimization for global optimization problems. 2021;:169.

[20] Altay O s m a n, Altay E l i f V a r o l. A novel hybrid multilayer perceptron neural network with improved grey wolf optimizer. Neural Computing and Applications. 2023;35(1):529–56.

[21] Lai X, Tu Y, Yan B, Wu L, Liu X. A Method for Predicting Ground Pressure in Meihua g Coal Mine Based on Improved BP Neural Network by Immune Algorithm-Particle Swarm Optimization. Processes. 2024;12(1):147.

[22] Luo Q, et al. Using spotted hyena optimizer for training feedforward neural networks. Cognitive Systems Research. 2021;65:1–16.

[23] Askari Q, Younas I. Political optimizer based feedforward neural network for classification and function approximation. Neural Processing Letters. 2021;53(1):429–58.

[24] Bashkandi AH, Hasanzade A, et al. Combination of political optimizer, particle swarm optimizer, and convolutional neural network for brain tumor detection. Biomedical Signal Processing and Control. 2023;81:104434.

[25] Chechkin AV, Metzler R, Klafter J, Gonchar V. Introduction to the theory of Lévy flights. Anomalous transport: foundations and applications. Wiley. 2008. p. 129–62.

[26] Mitra S, Acharyya S. Perturbation and repository based diversified cuckoo search in reconstruction of gene regulatory network: a new cuckoo search approach. Journal of Computational Science. 2022;60:101600.

[27] Li J, et al. Survey of lévy flight-based metaheuristics for optimization. Mathematics. 2022;10(15):2785.

[28] Padash A, et al. Asymmetric Lévy flights are more efficient in random search. Fractal and Fractional. 2022;6(5):260.

[29] Wang SM, et al. Transient electromagnetic method inversion based on Lévy flight-particle swarm optimization. Chinese Journal of Geophysics. 2022;65(4):1482–93.

[30] Balakrishnan K, et al. Improved equilibrium optimization based on Levy flight approach for feature selection. Evolving Systems. 2023;14(4):735–46.

[31] Zhou Z, Li F, Zhu H. An improved genetic algorithm using greedy strategy toward task scheduling optimization in cloud environments. Neural Computing and Applications. 2020;32:1531–41.

[32] Mahdavi S, Rahnamayan S, Deb K. Opposition based learning: A literature review. Swarm and evolutionary computation. 2018;39:1–23.

[33] Yao X, Liu Y, Lin G. Evolutionary programming made faster. IEEE Trans Evol Comput. 1999;3:82–102.

[34] Li J, Sun K. Pressure vessel design problem using improved gray wolf optimizer based on Cauchy distribution. Applied Sciences. 2023;13(22):12290.

[35] Kaur S, Awasthi LK, Sangal AL, Dhiman G. Tunicate swarm algorithm: A new bio-inspired based metaheuristic paradigm for global optimization. Engineering Applications of Artificial Intelligence. 2020;90:103541.

[36] Jia H, Peng X, Lang C. Remora optimization algorithm. Expert Systems with Applications. 2021;185:115665.

[37] Xue J, Shen B. Dung beetle optimizer: a new meta-heuristic algorithm for global optimization. J Supercomput. 2023;79:7305–36. Ahmadianfar I, Heidari AA, Gandomi AH, Chu X, Chen H. RUN beyond the metaphor: An efficient optimization algorithm based on Runge Kutta method. Expert Systems with Applications. 2021;181:115079.

[38] Chopra N, Mohsin Ansari M. Golden jackal optimization: A novel nature-inspired optimizer for engineering applications. Expert Systems with Applications. 2022;198:116924. doi: 10.1016/j.eswa.2022.116924

[39] Houssein EH, Oliva D, Samee NA, Mahmoud NF, Emam MM. Liver Cancer Algorithm: A novel bio-inspired optimizer. Comput Biol Med. 2023;165:107389. doi: 10.1016/j.compbiomed.2023.107389 37678138

[40] Gao Y. PID-based search algorithm: A novel metaheuristic algorithm based on PID algorithm. Expert Systems With Applications. 2023;232:120886.

[41] Wang J, Wang W, Hu X, Qiu L, Zang H. Black-winged kite algorithm: a nature-inspired meta-heuristic for solving benchmark functions and engineering problems. Artif Intell Rev. 2024;57(4). doi: 10.1007/s10462-024-10723-4

[42] Bi C, Tian Q, Chen H. Optimizing a multi-layer perceptron based on an improved gray wolf algorithm to identify plant diseases. Mathematics. 2023;11(15):3312.

[43] Landgrebe TCW, Duin RPW. Efficient multiclass ROC approximation by decomposition via confusion matrix perturbation analysis. IEEE Trans Pattern Anal Mach Intell. 2008;30(5):810–22. doi: 10.1109/TPAMI.2007.70740 18369251

[44] Mohamed AAA, Hançerlioğullari A, Rahebi J, Ray MK, Roy S. Colon Disease Diagnosis with Convolutional Neural Network and Grasshopper Optimization Algorithm. Diagnostics (Basel). 2023;13(10):1728. doi: 10.3390/diagnostics13101728 37238212 PMC10217543

[45] Hamel L. Model assessment with ROC curves. Encyclopedia of Data Warehousing and Mining. Second ed. IGI Global. 2009. p. 1316–23.

[46] Islam MM, Ferdousi R, Rahman S. Likelihood prediction of diabetes at early stage using data mining techniques. Computer Vision and Machine Intelligence in Medical Image Analysis. Singapore: Springer. 2020. p. 113–25.

[47] Strack B, DeShazo JO, Gennings C, Olmo JL, Ventura S, Cios KJ, et al. Impact of HbA1c measurement on hospital readmission rates: analysis of 70,000 clinical database patient records. BioMed Research International. 2014;2014. 10.1155/2014/781670 PMC3996476 24804245

[48] Congdon P, Lloyd P. Estimating small area diabetes prevalence in the US using the behavioral risk factor surveillance system. Journal of Data Science. 2010;8(2):235–52.

[49] Ahmed TM. Using data mining to develop model for classifying diabetic patient control level based on historical medical records. Journal of Theoretical and Applied Information Technology. 2016;87(2):316.

[50] Chang V, Ganatra MA, Hall K. An assessment of machine learning models and algorithms for early prediction and diagnosis of diabetes using health indicators. Healthcare Analytics. 2022;2:100118.

[51] Aure MR, Ragle M, et al. Crosstalk between microRNA expression and DNA methylation drives the hormone-dependent phenotype of breast cancer. Genome medicine. 2021;13(1):72. 33926515 10.1186/s13073-021-00880-4 PMC8086068

[52] Forconi F, Ashton-Key M, Meakin N. BCL2 inhibition in refractory hairy-cell leukemia. New England Journal of Medicine. 2023;388(21):2010–2. 37224205 10.1056/NEJMc2215613

[53] Nishiyama A, Nakanishi M. Navigating the DNA methylation landscape of cancer. Trends Genet. 2021;37(11):1012–27. doi: 10.1016/j.tig.2021.05.002 34120771

[54] Amirsadri S, Mousavirad SJ, Ebrahimpour-Komleh H. A Levy flight-based grey wolf optimizer combined with back-propagation algorithm for neural network training. Neural Computing and Applications. 2018;30:3707–20.

[55] Afreen S, Bhurjee AK, Aziz RM. Feature selection using game Shapley improved grey wolf optimizer for optimizing cancer classification. Knowledge and Information Systems. 2025;:1–32.

[56] Sharma A, Kumar P, Ben D. Improved GA based clustering with a new selection method for categorical dental data. Swarm optimization for biomedical applications. CRC Press. 2025. p. 172–92.

[57] Afreen S, Bhurjee AK, Aziz RM. Cancer classification using RNA sequencing gene expression data based on Game Shapley local search embedded binary social ski-driver optimization algorithms. Microchemical Journal. 2024;205:111280.

[58] Joshi AA, Aziz RM. Soft computing techniques for cancer classification of gene expression microarray data: A three-phase hybrid approach. Computational intelligence for data analysis. Bentham Science Publishers. 2024. p. 92–113.

[59] Afreen S, Kumar Bhurjee A, Musheer Aziz R. Study of optimality strategies for two-person game model under interval uncertainty. In: International conference on soft computing for problem-solving, Singapore, 2023. 45–60.

[60] Tomczak K, Czerwińska P, Wiznerowicz M. Review the cancer genome atlas (TCGA): an immeasurable source of knowledge. Contemporary Oncology/Współczesna Onkologia. 2015;2015(1):68–77. 10.5114/wo.2014.47136 PMC4322527 25691825

