# Supplementary material for: Neural network prediction model based on Levy flight and natural biomimetic technology for its application in cancer prediction
Source: PLoS One. 2025 Jun 25;20(6):e0326874. doi: 10.1371/journal.pone.0326874 (PMC12193836; doi:10.1371/journal.pone.0326874)
Supplement: S1 Table — (DOCX) [file pone.0326874.s001.docx]

**Supplementary Table S1. Calculation of Confidence Intervals (CIs) for Predictive Performance Metrics on Validation Sets**

|  |  |  |  |  |  |
| --- | --- | --- | --- | --- | --- |
|  |  | accuracy |  |  |  |
| dataset | moel | average - accuracy | lb（95% CI）- accuracy | ub（95% CI）- accuracy | std - accuracy |
| 5.1 | GWO | 0.923 | 0.899 | 0.947 | 0.192 |
| 5.1 | LGWO | 0.92 | 0.88 | 0.97 | 0.35 |
| 5.2 | GWO | 0.991 | 0.99 | 0.992 | 0.0009 |
| 5.2 | LGWO | 0.99 | 0.99 | 0.99 | 0 |
| 5.3 | GWO | 0.99 | 0.99 | 0.99 | 0.003 |
| 5.3 | LGWO | 0.99 | 0.99 | 0.99 | 0.002 |
| 5.4.1 | GWO | 0.64 | 0.62 | 0.66 | 0.01 |
| 5.4.1 | LGWO | 0.64 | 0.63 | 0.66 | 0.01 |
| 5.4.2 | GWO | 0.65 | 0.64 | 0.67 | 0.01 |
| 5.4.2 | LGWO | 0.65 | 0.65 | 0.66 | 0.004 |
| 5.4.3 | GWO | 0.66 | 0.64 | 0.69 | 0.02 |
| 5.4.3 | LGWO | 0.66 | 0.633 | 0.7 | 0.02 |
|  |  | recall |  |  |  |
| dataset | moel | average - recall | lb（95% CI）- recall | ub（95% CI）- recall | std - recall |
| 5.1 | GWO | 0.91 | 0.86 | 0.97 | 0.04 |
| 5.1 | LGWO | 0.93 | 0.83 | 1 | 0.08 |
| 5.2 | GWO | 1 | 1 | 1 | 0 |
| 5.2 | LGWO | 1 | 1 | 1 | 0 |
| 5.3 | GWO | 1 | 1 | 1 | 0.007 |
| 5.3 | LGWO | 1 | 1 | 1 | 0.003 |
| 5.4.1 | GWO | 0.7 | 0.61 | 0.79 | 0.07 |
| 5.4.1 | LGWO | 0.71 | 0.62 | 0.8 | 0.07 |
| 5.4.2 | GWO | 0.63 | 0.59 | 0.68 | 0.03 |
| 5.4.2 | LGWO | 0.61 | 0.58 | 0.65 | 0.03 |
| 5.4.3 | GWO | 0.61 | 0.58 | 0.64 | 0.02 |
| 5.4.3 | LGWO | 0.62 | 0.6 | 0.65 | 0.02 |
|  |  | precision |  |  |  |
| dataset | moel | average - precision | lb（95% CI）- precision | ub（95% CI）- precision | std -precision |
| 5.1 | GWO | 0.89 | 0.87 | 0.91 | 0.14 |
| 5.1 | LGWO | 0.89 | 0.85 | 0.92 | 0.03 |
| 5.2 | GWO | 0.96 | 0.96 | 0.97 | 0.003 |
| 5.2 | LGWO | 0.96 | 0.96 | 0.97 | 0.003 |
| 5.3 | GWO | 0.96 | 0.96 | 0.97 | 0.004 |
| 5.3 | LGWO | 0.97 | 0.96 | 0.97 | 0.005 |
| 5.4.1 | GWO | 0.68 | 0.63 | 0.72 | 0.03 |
| 5.4.1 | LGWO | 0.68 | 0.63 | 0.72 | 0.04 |
| 5.4.2 | GWO | 0.68 | 0.66 | 0.71 | 0.02 |
| 5.4.2 | LGWO | 0.69 | 0.67 | 0.71 | 0.02 |
| 5.4.3 | GWO | 0.63 | 0.59 | 0.67 | 0.03 |
| 5.4.3 | LGWO | 0.62 | 0.58 | 0.67 | 0.03 |
|  |  | F1-score |  |  |  |
| dataset | moel | average - F1-score | lb（95% CI）- F1-score | ub（95% CI）- F1-score | std - F1-score |
| 5.1 | GWO | 0.9 | 0.88 | 0.93 | 0.19 |
| 5.1 | LGWO | 0.91 | 0.86 | 0.96 | 0.04 |
| 5.2 | GWO | 0.98 | 0.97 | 0.98 | 0 |
| 5.2 | LGWO | 0.98 | 0.98 | 0.98 | 0.001 |
| 5.3 | GWO | 0.98 | 0.97 | 0.98 | 0.004 |
| 5.3 | LGWO | 0.98 | 0.98 | 0.98 | 0.002 |
| 5.4.1 | GWO | 0.69 | 0.65 | 0.72 | 0.02 |
| 5.4.1 | LGWO | 0.69 | 0.67 | 0.71 | 0.02 |
| 5.4.2 | GWO | 0.66 | 0.64 | 0.68 | 0.02 |
| 5.4.2 | LGWO | 0.65 | 0.63 | 0.67 | 0.02 |
| 5.4.3 | GWO | 0.62 | 0.59 | 0.64 | 0.02 |
| 5.4.3 | LGWO | 0.62 | 0.6 | 0.65 | 0.02 |
|  |  | AUC |  |  |  |
| dataset | moel | average - AUC | lb（95% CI）- AUC | ub（95% CI）- AUC | std -AUC |
| 5.1 | GWO | 0.97 | 0.96 | 0.99 | 0.14 |
| 5.1 | LGWO | 0.95 | 0.9 | 1 | 0.04 |
| 5.2 | GWO | 1 | 1 | 1 | 0 |
| 5.2 | LGWO | 1 | 1 | 1 | 0 |
| 5.3 | GWO | 1 | 1 | 1 | 0.002 |
| 5.3 | LGWO | 1 | 1 | 1 | 0.01 |
| 5.4.1 | GWO | 0.69 | 0.67 | 0.71 | 0.02 |
| 5.4.1 | LGWO | 0.7 | 0.67 | 0.71 | 0.02 |
| 5.4.2 | GWO | 0.71 | 0.7 | 0.72 | 0.008 |
| 5.4.2 | LGWO | 0.72 | 0.71 | 0.72 | 0.01 |
| 5.4.3 | GWO | 0.72 | 0.69 | 0.74 | 0.02 |
| 5.4.3 | LGWO | 0.72 | 0.69 | 0.76 | 0.02 |
