# Supplementary material for: Neural network prediction model based on Levy flight and natural biomimetic technology for its application in cancer prediction
Source: PLoS One. 2025 Jun 25;20(6):e0326874. doi: 10.1371/journal.pone.0326874 (PMC12193836; doi:10.1371/journal.pone.0326874)
Supplement: S2.1 Table — (DOCX) [file pone.0326874.s002.docx]

**Supplementary Table S2.1 Performance metrics for another class**

| **performance metrics for another class** | | | | | |
| --- | --- | --- | --- | --- | --- |
| GWO | accuracy | recall | precision | F1-score | AUC |
| dataset5.1 | 0.942308 | 0.943662 | 0.971014 | 0.957143 | 0.991464 |
| dataset5.2 | 0.9911 | 0.9884 | 1 | 0.9942 | 0.995701 |
| dataset5.3 | 0.9896 | 0.9994 | 0.958 | 0.9782 | 0.994728 |
| dataset5.4.1 | 0.6478 | 0.5461 | 0.6126 | 0.5774 | 0.688848 |
| dataset5.4.2 | 0.6461 | 0.5662 | 0.6432 | 0.6023 | 0.721396 |
| dataset5.4.3 | 0.6578 | 0.7802 | 0.6637 | 0.7173 | 0.724215 |
| LGWO | accuracy | recall | precision | F1-score | AUC |
| dataset5.1 | 0.961538 | 0.985915 | 0.958904 | 0.972222 | 0.99061 |
| dataset5.2 | 0.9917 | 0.9894 | 0.9999 | 0.9946 | 0.996226 |
| dataset5.3 | 0.9898 | 0.9998 | 0.9582 | 0.9785 | 0.995236 |
| dataset5.4.1 | 0.6396 | 0.5834 | 0.5924 | 0.5879 | 0.693509 |
| dataset5.4.2 | 0.6587 | 0.6806 | 0.6288 | 0.6537 | 0.719453 |
| dataset5.4.3 | 0.6667 | 0.6881 | 0.7055 | 0.6967 | 0.716287 |
