# Supplementary material for: Neural network prediction model based on Levy flight and natural biomimetic technology for its application in cancer prediction
Source: PLoS One. 2025 Jun 25;20(6):e0326874. doi: 10.1371/journal.pone.0326874 (PMC12193836; doi:10.1371/journal.pone.0326874)
Supplement: S2.2 Table — (DOCX) [file pone.0326874.s003.docx]

**Supplementary Table S2.2 Performance Metrics for Macro Average**

| **performance metrics for Macro Average** | | | | | |
| --- | --- | --- | --- | --- | --- |
| GWO | accuracy | recall | precision | F1-score | AUC |
| dataset5.1 | 0.93 | 0.93 | 0.93 | 0.93 | 0.96 |
| dataset5.2 | 0.99 | 1 | 0.98 | 0.99 | 1 |
| dataset5.3 | 0.99 | 0.99 | 0.96 | 0.98 | 0.99 |
| dataset5.4.1 | 0.65 | 0.63 | 0.65 | 0.63 | 0.69 |
| dataset5.4.2 | 0.65 | 0.6 | 0.66 | 0.63 | 0.72 |
| dataset5.4.3 | 0.66 | 0.69 | 0.64 | 0.67 | 0.71 |
| LGWO | accuracy | recall | precision | F1-score | AUC |
| dataset5.1 | 0.94 | 0.96 | 0.92 | 0.94 | 0.97 |
| dataset5.2 | 0.99 | 1 | 0.99 | 0.99 | 1 |
| dataset5.3 | 0.99 | 1 | 0.96 | 0.98 | 0.99 |
| dataset5.4.1 | 0.64 | 0.65 | 0.64 | 0.64 | 0.7 |
| dataset5.4.2 | 0.66 | 0.65 | 0.66 | 0.65 | 0.72 |
| dataset5.4.3 | 0.67 | 0.66 | 0.67 | 0.66 | 0.72 |
