# Supplementary material for: Neural network prediction model based on Levy flight and natural biomimetic technology for its application in cancer prediction
Source: PLoS One. 2025 Jun 25;20(6):e0326874. doi: 10.1371/journal.pone.0326874 (PMC12193836; doi:10.1371/journal.pone.0326874)
Supplement: S3 Table — (DOCX) [file pone.0326874.s005.docx]

**Supplementary Table S3. Wilcoxon signed-rank test results for GWO-BP vs. LGWO-BP comparison**

|  |  |  | Wilcoxon |  |  |  |
| --- | --- | --- | --- | --- | --- | --- |
|  | dataset5.1 |  |  |  |  |  |
| GWO/LGWO | accuracy |  | recall | precision | F1-score | AUC |
|  | 0.713 |  | 0.5 | 0.686 | 0.686 | 0.345 |
|  | dataset5.2 |  |  |  |  |  |
| GWO/LGWO | accuracy |  | recall | precision | F1-score | AUC |
|  | 0.414 |  | 0.18 | 1 | 0.414 | 0.893 |
|  | dataset5.3 |  |  |  |  |  |
| GWO/LGWO | accuracy |  | recall | precision | F1-score | AUC |
|  | 0.144 |  | 0.138 | 0.686 | 0.225 | 0.225 |
|  | dataset5.4.2 |  |  |  |  |  |
| GWO/LGWO | accuracy |  | recall | precision | F1-score | AUC |
|  | 0.5 |  | 0.345 | 0.5 | 0.5 | 0.686 |
|  | dataset5.4.1 |  |  |  |  |  |
| GWO/LGWO | accuracy |  | recall | precision | F1-score | AUC |
|  | 0.686 |  | 0.5 | 0.5 | 0.345 | 0.686 |
|  | dataset5.4.3 |  |  |  |  |  |
| GWO/LGWO | accuracy |  | recall | precision | F1-score | AUC |
|  | 0.893 |  | 0.225 | 0.893 | 0.225 | 0.225 |
