# Supplementary material for: Neural network prediction model based on Levy flight and natural biomimetic technology for its application in cancer prediction
Source: PLoS One. 2025 Jun 25;20(6):e0326874. doi: 10.1371/journal.pone.0326874 (PMC12193836; doi:10.1371/journal.pone.0326874)
Supplement: S4 Table — (DOCX) [file pone.0326874.s006.docx]

**Supplementary Table S4. Cohen’s d effect sizes for GWO-BP vs. LGWO-BP comparison**

|  | Cohen’s d | |  |  |  |
| --- | --- | --- | --- | --- | --- |
|  | dataset5.1 |  |  |  |  |
| GWO/LGWO | accuracy | recall | precision | F1-score | AUC |
|  | 0.03 | 0.07 | 0.03 | 0.04 | 0.03 |
|  | dataset5.2 |  |  |  |  |
| GWO/LGWO | accuracy | recall | precision | F1-score | AUC |
|  | 0.001 | 0 | 0.003 | 0.002 | 0.001 |
|  | dataset5.3 |  |  |  |  |
| GWO/LGWO | accuracy | recall | precision | F1-score | AUC |
|  | 0.009 | 0.006 | 0.005 | 0.006 | 0.002 |
|  | dataset5.4.2 |  |  |  |  |
| GWO/LGWO | accuracy | recall | precision | F1-score | AUC |
|  | 0.01 | 0.03 | 0.02 | 0.02 | 0.008 |
|  | dataset5.4.1 |  |  |  |  |
| GWO/LGWO | accuracy | recall | precision | F1-score | AUC |
|  | 0.02 | 0.07 | 0.04 | 0.02 | 0.02 |
|  | dataset5.4.3 |  |  |  |  |
| GWO/LGWO | accuracy | recall | precision | F1-score | AUC |
|  | 0.02 | 0.02 | 0.03 | 0.02 | 0.03 |
