# Supplementary material for: Neural network prediction model based on Levy flight and natural biomimetic technology for its application in cancer prediction
Source: PLoS One. 2025 Jun 25;20(6):e0326874. doi: 10.1371/journal.pone.0326874 (PMC12193836; doi:10.1371/journal.pone.0326874)
Supplement: S5 Table — (DOCX) [file pone.0326874.s007.docx]

**Supplementary Table S5. Brier score comparison between GWO-BP and LGWO-BP**

| Brier score | GWO | LGWO |
| --- | --- | --- |
| dataset5.1 | 0.49 | 0.5287 |
| dataset5.2 | 0.7616 | 0.7616 |
| dataset5.3 | 0.3324 | 0.3229 |
| dataset5.4.1 | 0.2597 | 0.2576 |
| dataset5.4.2 | 0.2079 | 0.2257 |
| dataset5.4.3 | 0.3585 | 0.3492 |
