# Supplementary material for: Neural network prediction model based on Levy flight and natural biomimetic technology for its application in cancer prediction
Source: PLoS One. 2025 Jun 25;20(6):e0326874. doi: 10.1371/journal.pone.0326874 (PMC12193836; doi:10.1371/journal.pone.0326874)
Supplement: S6 Table — (DOCX) [file pone.0326874.s008.docx]

**Supplementary Table S6. 5-fold cross-validation performance comparison between GWO-BP and LGWO-BP**

| dataset5.1 | | | | | | | | | | | |
| --- | --- | --- | --- | --- | --- | --- | --- | --- | --- | --- | --- |
| GWO | accuracy | recall | precision | F1-score | AUC | LGWO | accuracy | recall | precision | F1-score | AUC |
| fold1 | 0.95 | 0.97 | 0.89 | 0.93 | 0.99 |  | 0.95 | 0.94 | 0.91 | 0.93 | 0.98 |
| fold2 | 0.91 | 0.93 | 0.87 | 0.90 | 0.99 |  | 0.92 | 1.00 | 0.84 | 0.91 | 0.91 |
| fold3 | 0.90 | 0.86 | 0.90 | 0.88 | 0.96 |  | 0.87 | 0.80 | 0.88 | 0.83 | 0.90 |
| fold4 | 0.91 | 0.88 | 0.90 | 0.89 | 0.96 |  | 0.94 | 0.95 | 0.90 | 0.93 | 0.98 |
| fold5 | 0.93 | 0.93 | 0.90 | 0.91 | 0.97 |  | 0.94 | 0.95 | 0.90 | 0.93 | 0.98 |
| dataset5.2 | | | | | | | | | | | |
| GWO | accuracy | recall | precision | F1-score | AUC | LGWO | accuracy | recall | precision | F1-score | AUC |
| fold1 | 0.99 | 1.00 | 0.97 | 0.98 | 1.00 | fold1 | 0.99 | 1.00 | 0.97 | 0.98 | 1.00 |
| fold2 | 0.99 | 1.00 | 0.96 | 0.98 | 1.00 | fold2 | 0.99 | 1.00 | 0.96 | 0.98 | 1.00 |
| fold3 | 0.99 | 1.00 | 0.96 | 0.98 | 1.00 | fold3 | 0.99 | 1.00 | 0.96 | 0.98 | 1.00 |
| fold4 | 0.99 | 1.00 | 0.96 | 0.98 | 1.00 | fold4 | 0.99 | 1.00 | 0.96 | 0.98 | 1.00 |
| fold5 | 0.99 | 1.00 | 0.96 | 0.98 | 0.99 | fold5 | 0.99 | 1.00 | 0.96 | 0.98 | 1.00 |
| dataset5.3 | | | | | | | | | | | |
| GWO | accuracy | recall | precision | F1-score | AUC | LGWO | accuracy | recall | precision | F1-score | AUC |
| fold1 | 0.99 | 1.00 | 0.96 | 0.98 | 1.00 | fold1 | 0.99 | 1.00 | 0.97 | 0.98 | 0.99 |
| fold2 | 0.99 | 1.00 | 0.96 | 0.98 | 1.00 | fold2 | 0.99 | 1.00 | 0.96 | 0.98 | 1.00 |
| fold3 | 0.99 | 1.00 | 0.96 | 0.98 | 0.99 | fold3 | 0.99 | 1.00 | 0.96 | 0.98 | 1.00 |
| fold4 | 0.99 | 1.00 | 0.96 | 0.98 | 0.99 | fold4 | 0.99 | 1.00 | 0.97 | 0.98 | 1.00 |
| fold5 | 0.99 | 1.00 | 0.96 | 0.98 | 0.99 | fold5 | 0.99 | 1.00 | 0.96 | 0.98 | 0.99 |
| dataset5.4.1 | | | | | | | | | | | |
| GWO | accuracy | recall | precision | F1-score | AUC | LGWO | accuracy | recall | precision | F1-score | AUC |
| fold1 | 0.64 | 0.69 | 0.70 | 0.69 | 0.69 | fold1 | 0.64 | 0.64 | 0.71 | 0.68 | 0.69 |
| fold2 | 0.67 | 0.74 | 0.70 | 0.72 | 0.71 | fold2 | 0.66 | 0.73 | 0.69 | 0.71 | 0.71 |
| fold3 | 0.63 | 0.79 | 0.62 | 0.69 | 0.67 | fold3 | 0.63 | 0.82 | 0.61 | 0.70 | 0.67 |
| fold4 | 0.63 | 0.60 | 0.71 | 0.65 | 0.68 | fold4 | 0.64 | 0.65 | 0.70 | 0.67 | 0.69 |
| fold5 | 0.64 | 0.67 | 0.67 | 0.67 | 0.70 | fold5 | 0.65 | 0.73 | 0.66 | 0.69 | 0.70 |
| dataset5.4.2 | | | | | | | | | | | |
| GWO | accuracy | recall | precision | F1-score | AUC | LGWO | accuracy | recall | precision | F1-score | AUC |
| fold1 | 0.67 | 0.69 | 0.67 | 0.68 | 0.72 | fold1 | 0.66 | 0.61 | 0.69 | 0.65 | 0.73 |
| fold2 | 0.65 | 0.64 | 0.68 | 0.66 | 0.72 | fold2 | 0.65 | 0.57 | 0.71 | 0.63 | 0.71 |
| fold3 | 0.63 | 0.61 | 0.69 | 0.65 | 0.70 | fold3 | 0.65 | 0.63 | 0.70 | 0.66 | 0.71 |
| fold4 | 0.66 | 0.61 | 0.71 | 0.66 | 0.71 | fold4 | 0.65 | 0.65 | 0.69 | 0.67 | 0.71 |
| fold5 | 0.65 | 0.61 | 0.66 | 0.64 | 0.71 | fold5 | 0.65 | 0.59 | 0.67 | 0.63 | 0.71 |
| dataset5.4.3 | | | | | | | | | | | |
| GWO | accuracy | recall | precision | F1-score | AUC | LGWO | accuracy | recall | precision | F1-score | AUC |
| fold1 | 0.65 | 0.60 | 0.59 | 0.59 | 0.70 | fold1 | 0.64 | 0.64 | 0.57 | 0.60 | 0.70 |
| fold2 | 0.66 | 0.59 | 0.62 | 0.61 | 0.71 | fold2 | 0.67 | 0.61 | 0.64 | 0.62 | 0.73 |
| fold3 | 0.65 | 0.63 | 0.60 | 0.62 | 0.71 | fold3 | 0.65 | 0.59 | 0.61 | 0.60 | 0.72 |
| fold4 | 0.70 | 0.63 | 0.66 | 0.64 | 0.76 | fold4 | 0.71 | 0.65 | 0.67 | 0.66 | 0.76 |
| fold5 | 0.67 | 0.58 | 0.67 | 0.62 | 0.71 | fold5 | 0.65 | 0.63 | 0.62 | 0.62 | 0.71 |
