# Supplementary material for: Neural network prediction model based on Levy flight and natural biomimetic technology for its application in cancer prediction
Source: PLoS One. 2025 Jun 25;20(6):e0326874. doi: 10.1371/journal.pone.0326874 (PMC12193836; doi:10.1371/journal.pone.0326874)
Supplement: S7 Table — (DOCX) [file pone.0326874.s009.docx]

**Supplementary Table S7. Performance Comparison Across Different Hidden Layer Sizes.**

| hidden size=4 | |  |  |  |  |
| --- | --- | --- | --- | --- | --- |
|  |  | GWO |  |  |  |
| dataset | accuracy | recall | precision | F1-score | AUC |
| 5.1 | 0.89103 | 0.91667 | 0.8209 | 0.86614 | 0.96 |
| 5.2 | 0.99116 | 0.99986 | 0.9631 | 0.98113 | 1 |
| 5.4.1 | 0.63856 | 0.70772 | 0.66667 | 0.68658 | 0.69 |
| 5.4.2 | 0.65217 | 0.60653 | 0.69451 | 0.64754 | 0.71 |
| 5.4.3 | 0.66932 | 0.58408 | 0.63943 | 0.6105 | 0.72 |
|  |  | LGWO |  |  |  |
| dataset | accuracy | recall | precision | F1-score | AUC |
| 5.1 | 0.89744 | 0.85 | 0.87931 | 0.86441 | 0.94 |
| 5.2 | 0.99116 | 1 | 0.96297 | 0.98113 | 1 |
| 5.4.1 | 0.65501 | 0.74265 | 0.67389 | 0.7066 | 0.7 |
| 5.4.2 | 0.66633 | 0.66027 | 0.69215 | 0.67583 | 0.72 |
| 5.4.3 | 0.66049 | 0.63881 | 0.6126 | 0.62543 | 0.72 |
| hidden size=8 | |  |  |  |  |
|  |  | GWO |  |  |  |
| dataset | accuracy | recall | precision | F1-score | AUC |
| 5.1 | 0.89744 | 0.91667 | 0.83333 | 0.87302 | 0.97 |
| 5.2 | 0.99122 | 1 | 0.96323 | 0.98127 | 1 |
| 5.4.1 | 0.63136 | 0.72151 | 0.65471 | 0.68649 | 0.67 |
| 5.4.2 | 0.64257 | 0.59405 | 0.68549 | 0.6365 | 0.7 |
| 5.4.3 | 0.65121 | 0.49254 | 0.63871 | 0.55618 | 0.71 |
|  |  | LGWO |  |  |  |
| dataset | accuracy | recall | precision | F1-score | AUC |
| 5.1 | 0.94231 | 0.98333 | 0.8806 | 0.92913 | 0.97 |
| 5.2 | 0.99053 | 1 | 0.96047 | 0.97983 | 1 |
| 5.4.1 | 0.63805 | 0.65717 | 0.68356 | 0.6701 | 0.69 |
| 5.4.2 | 0.65268 | 0.69002 | 0.6639 | 0.67671 | 0.71 |
| 5.4.3 | 0.66711 | 0.67463 | 0.61357 | 0.64265 | 0.73 |
| hidden size=10 | |  |  |  |  |
|  |  | GWO |  |  |  |
| dataset | accuracy | recall | precision | F1-score | AUC |
| 5.1 | 0.94231 | 0.98333 | 0.8806 | 0.92913 | 0.99 |
| 5.2 | 0.99125 | 1 | 0.96336 | 0.98134 | 1 |
| 5.4.1 | 0.63342 | 0.76471 | 0.64546 | 0.70004 | 0.68 |
| 5.4.2 | 0.6451 | 0.61708 | 0.6797 | 0.64688 | 0.71 |
| 5.4.3 | 0.64636 | 0.46965 | 0.63784 | 0.54097 | 0.7 |
|  |  | LGWO |  |  |  |
| dataset | accuracy | recall | precision | F1-score | AUC |
| 5.1 | 0.91026 | 0.95 | 0.83824 | 0.89062 | 0.96 |
| 5.2 | 0.99158 | 1 | 0.96469 | 0.98203 | 1 |
| 5.4.1 | 0.65039 | 0.74081 | 0.66944 | 0.70332 | 0.7 |
| 5.4.2 | 0.67897 | 0.64012 | 0.71953 | 0.6775 | 0.72 |
| 5.4.3 | 0.66225 | 0.599 | 0.62448 | 0.61148 | 0.72 |
